# Embolic Strokes of Unknown Source and Cryptogenic Stroke: Implications in Clinical Practice

**DOI:** 10.3389/fneur.2016.00037

**Published:** 2016-03-21

**Authors:** Amre Nouh, Mohammed Hussain, Tapan Mehta, Shadi Yaghi

**Affiliations:** ^1^Hartford Hospital, University of Connecticut, Hartford, CT, USA; ^2^Brown University, Providence, RI, USA

**Keywords:** cryptogenic stroke, embolic stroke of undetermined source, patent foramen ovale, paroxysmal atrial fibrillation, hypercoagulable state, cardiac monitoring, aortic arch atherosclerosis

## Abstract

Up to a third of strokes are rendered cryptogenic or of undetermined etiology. This number is specifically higher in younger patients. At times, inadequate diagnostic workups, multiple causes, or an under-recognized etiology contributes to this statistic. Embolic stroke of undetermined source, a new clinical entity particularly refers to patients with embolic stroke for whom the etiology of embolism remains unidentified despite through investigations ruling out established cardiac and vascular sources. In this article, we review current classification and discuss important clinical considerations in these patients; highlighting cardiac arrhythmias and structural abnormalities, patent foramen ovale, paradoxical sources, and potentially under-recognized, vascular, inflammatory, autoimmune, and hematologic sources in relation to clinical practice.

## Definition, Classification, and Risk

Approximately one-third of all ischemic strokes are of undetermined etiology and are more prevalent among young adults (1–3). The term “cryptogenic stroke” (CS) has been extensively used in the literature to describe this subtype. However, this does not take into account the extent and quality of the investigation performed or classification system used. In the TOAST classification system, stroke of undetermined cause may refer to a stroke with incomplete workup, more than one potential cause, or indeed no determined etiology after investigations are complete (4). In the ASCO classification, cause is completely unknown when stroke subtyping does not confer to atherosclerosis (A), small vessel disease (S), cardiac disease (C), or other cause (O) (5). This allows for potential misclassification and overrepresentation of CS using these systems. In some instances, young stroke patients have been classified as cardioembolic based on the finding of a patent foramen ovale (PFO) (6). Furthermore, patients with few traditional risk factors may be classified as cryptogenic. However, clinical, radiographic, and risk factor profiles vary. Thorough etiological investigations and appropriate classification are needed to best categorize these patients.

In 2014, the term *Embolic stroke of undetermined source* (ESUS) was coined by the CS/ESUS international working group (7). ESUS refers to non-lacunar infarct (subcortical infarct ≤1.5 cm on CT or ≤2.0 cm on MRI) in the absence the following: extracranial or intracranial atherosclerosis causing >50% luminal stenosis in the artery supplying the ischemic region, major cardiomebolic sources [permanent or paroxysmal atrial fibrillation (AF), sustained atrial flutter, intracardiac thrombus, prosthetic cardiac valve, atrial myxoma or other cardiac tumors, mitral stenosis, myocardial infarction within the past 4 weeks, left ventricular (LV) ejection fraction <30%, valvular vegetation’s or infective endocarditis], and no other specific cause of stroke (e.g., dissection, arteritis, migraine/vasospasm, drug misuse). This allows a more comprehensive investigation and exclusion of presumed non-embolic stroke mechanisms during evaluation. Due to the novelty of this term, studies in the literature have mostly used the term CS, which may include patient fulfilling ESUS criteria.

Patients with CS, and especially the young, typically lack traditional stroke risk factors (3). Although recurrent stroke in patients with CS and ESUS varies, the risk remains high. In a recent population-based study including 2555 patients, the 10-year risk of recurrence in CS vs. non-cardioembolic stroke was 32 vs. 27% (*p* = 0.91). Moreover, compared with large and small vessel disease subtypes combined, patients with CS had no excess of minor-risk echocardiographic abnormalities, paroxysmal AF at baseline or new onset AF, or presumed cardioembolic events during follow-up (3). In the Athens Stroke Registry, stroke recurrence in ESUS patients was also high (29.0%) similar to recurrence risk of cardioembolic stroke (26%) and significantly higher as compared to all other non-cardioembolic stroke subtypes (8).

## Possible Etiologies of ESUS and CS

### Cardiac Atrial Fibrillation and Cryptogenic Stroke

Atrial fibrillation has been considered to be a precursor for left atrial stasis, thrombus formation, and subsequent embolism where treatment with anticoagulation therapy has been shown to be superior to antiplatelet therapy in reducing the embolic risk (9). Since AF can be paroxysmal, there has been growing evidence to support outpatient cardiac monitoring to increase the detection of AF in patients with CS, which culminated in two randomized trials proving an increase in detection rates with prolonged monitoring when compared to standard of care.

The EMBRACE trial randomized 572 patients with CS or transient ischemic attack to standard 24-h Holter monitor vs. 30-day outpatient event-triggered monitor. The primary outcome was 30 s of AF detected at 90 days. In this study, the detection rate was significantly higher in patients receiving 30 day monitoring compared to 24 h telemetry (16.1 vs. 3.2%, *P* < 0.001) (10). On the other hand, the CRYSTAL-AF study randomized 441 patients with CS or TIA to long-term monitoring with REVEAL implantable cardiac monitor (ICM) vs. standard of care. The primary outcome was first detection of AF at 6 months. AF was detected at a rate of 8.9% of patients in the ICM group, as compared with 1.4% in the control group, yielding a hazard ratio of 6.4 (95% CI, 1.9–21.7; *P* < 0.001). There was an incremental increase in the detection rate with time yielding a hazard ratio of 8.78 (3.47–22.19) at 3 years (11). The reason for differences in the detection rates between the two studies remains unclear. However, this may be due to differences in the study populations, stroke diagnostic evaluation, and inclusion and exclusion criteria. Results of these trials suggest a robust role for outpatient cardiac monitoring after a CS and the longer patients are monitored, the higher the yield of detecting AF. It remains unclear, however, what the optimal monitoring duration and modality is. The rate of AF detection varies between 2.7 and 30% depending on duration and modality of monitoring (Table 1) (12).

**Table 1:** **Cardiac monitoring for detection of atrial fibrillation in ESUS and CS**.

Type of monitoring	Setting	Invasive vs. non-invasive	Duration	Rate of detection of atrial fibrillation (%)
**Observational/retrospective studies**				
Admission ECG	Inpatient	Non-invasive	N/A	2.7
Inpatient continuous telemetry	Inpatient	Non-invasive	3–5 days	5.5–7.6
Holter monitor	Outpatient	Non-invasive	24–48 h	3.2–6.4
Mobile continuous outpatient telemetry	Outpatient	Non-invasive	21–30 days	16–25
Implantable loop recorders	Outpatient	Invasive	6–18 months	20–30
**Randomized clinical trials**				
Mobile continuous outpatient telemetry	Outpatient	Non-invasive	28 days	16.2
Implantable cardiac monitor	Outpatient	Invasive	6–36 months	9–30

Several predictors of AF detection in patients with CS have been identified. Older age (13) and prior embolic infarcts (13, 14) were the most robust clinical and radiological predictor of AF that were identified in studies. In addition, cardiac predictors included left atrial enlargement on echocardiogram and premature atrial complex on ECG or inpatient cardiac telemetry (15, 16). Based on current evidence, starting with the 30-day non-invasive cardiac monitoring is a reasonable first approach. If AF is not detected, prolonged monitoring with ICM may be considered.

#### Atrial “Cardiopathy” and Cryptogenic Stroke

For long, it has been proposed that the fibrillating left atrium is a precursor for thrombus formation and cardioembolism. Recent evidence, however, challenges the concept that AF by itself is the direct cause of thromboembolic events in these patients. The ASSERT study enrolled 2580 patients who were 65 years or older with a history of hypertension and without known AF who underwent cardiac pacemaker implantation and monitored them for 2.5 years. This study showed that subclinical AF was detected in approximately 10% of patients at 3 months and only 15% of them developed clinical AF. In addition, subclinical AF was predictive of ischemic stroke or systemic embolism risk (adjusted hazard ratio 2.50; 95% CI, 1.28–4.89; *P* = 0.008) (16). In this study, of the 51 patients who had ischemic stroke or systemic embolism, 26 (51%) had subclinical AF >6 min, and of these patients, 18 had AF detected prior to the event but only 4 patients had it in the 30 days preceding the event. The rest had AF for the first time after the embolic event. These findings and findings from another study (17) suggest lack of a temporal relationship between subclinical AF and embolic events, challenging the old concept that AF is the major determinant of embolic risk in patients with this dysrhythmia (16, 18). The findings rather suggest that AF may be a marker of atrial dysfunction (19) or “cardiopathy,” which in turn is the direct cause of embolic events in these patients.

Furthermore, there is growing evidence to suggest an association between biomarkers of atrial dysfunction or cardiopathy and ischemic stroke independent of AF. Electrocardiogram (ECG) parameters of left atrial dysfunction have been shown to be associated with ischemic stroke risk. In a study using a statewide administrative database, when compared to matched controls, patients with supraventricular tachycardia were shown to have a twofold increase in risk of ischemic stroke (hazard ratio, 2.10; 95% confidence interval, 1.69–2.62) (20, 21). In population-based cohorts, p-wave dispersion on ECG, another marker of left atrial dysfunction, was shown to be associated with ischemic stroke risk (22), especially those of embolic subtype (23).

Serum N-terminal pro-BNP is a biomarker that can be elevated in patients with heart failure and atrial stretch and predicts incident of AF (24). Several studies showed an association between serum NT-proBNP level and ischemic stroke risk (especially those of embolic subtype), a relationship independent of baseline or incident AF (16, 25, 26). In addition, left atrial enlargement on echocardiogram can lead to stasis, thrombus formation, and embolic events. Recently, an analysis from the Northern Manhattan Stroke Study showed an association between moderate to severe left atrial enlargement and recurrent embolic stroke independent of AF (21).

Since AF can be paroxysmal and difficult to diagnose even after prolonged monitoring and since recent studies suggest the lack of causality between AF and embolic events, it may be appealing to look for more permanent biomarkers in CS patients with an aim to evaluate left atrial function and understand the risk of stroke recurrence. A recent analysis from the SPOTRIAS data showed that biomarkers of atrial cardiopathy can be present in up to 65% of patients with CS and these biomarkers were associated with vascular risk factors and inversely associated with other potential stroke mechanisms such as PFO (27). Since long-term monitoring for AF was not performed routinely in these studies, it remains unclear whether or not this association is mediated by the presence of subclinical paroxysmal AF despite the relationship between biomarkers of atrial cardiopathy and stroke risk.

Patients with CS who have evidence of atrial cardiopathy may therefore constitute a group where anticoagulation therapy may be superior to antiplatelet therapy in reducing the risk of recurrent stroke. In fact, while the WARSS study did not show any benefit of warfarin over aspirin in reducing the 2-year risk of stroke or death, a *post hoc* analysis of WARSS showed that in patients with NT-proBNP >750 ng/dL, a biomarker of atrial cardiopathy, warfarin was superior to aspirin in reducing the risk of stroke or death at 2 years (28).

#### Patent Foramen Ovale

Patent foramen ovale is the most common congenital cardiac abnormality present in approximately 25% of the population (29) and responsible for up to 95% of right-to-left shunts (30). PFO prevalence among patients with CS are typically high with median prevalence of approximately 40% (31) and more commonly present in the young (31, 32). A meta-analysis of 23 studies evaluating CS and PFO’s concluded the odds of finding a PFO is 2.9 times higher in patients with CS as compared to control subjects (31). Several PFO characteristics with a higher probability for thromboembolism have been suggested. Atrial septal aneurysm has been most commonly reported; as incidence is higher among both older and younger patients with CS (33) Excursion of ≥10 mm of the interatrial septum during the cardiac cycle has been associated with increased risk of recurrent stroke (34). In addition, severe left-to-right shunt and large opening of PFO have also been proposed (35). However, these and other anatomic and physiologic findings such as shunting without Valsalva, Chiari network, and prominent Eustichian valves have shown inconsistent findings regarding stroke recurrence (34).

Patients are usually unaware of having a PFO until discovered during diagnostic evaluation for stroke etiology. Saline contrast injection or agitated saline mixed with air (also referred to as a “bubble study”) during TTE or TEE can detect a PFO if microbubbles are seen within the left atrial chamber within three cardiac cycles after right atrial opacification (29).

Directly establishing the PFO as the source of stroke is challenging. *In situ* thrombus formation and propensity for cardiac arrhythmias in these patients have been suggested mechanisms (36). Although cases of direct visualization of thrombus within the PFO have been reported (37), it is rarely observed. By far, paradoxical embolization is the most commonly proposed mechanism for stroke. Therefore, finding a proximal source such as a deep venous thrombus (DVT) is warranted, otherwise the association is rendered theoretical.

The reported frequency of DVT detection in patients with PFO and stroke has been 7.6–9.5% (32, 38, 39). Of major importance, in one study 80% of DVT’s detected were asymptomatic, emphasizing the need of ultrasound phlebography in patients with PFO and stroke of unknown source (32). With an advantage above ultrasound, magnetic resonance venography (MRV) can help detect isolated pelvic DVT’s. Although considered a rare entity, isolated pelvic thrombi have been seen in up to 20% of patients with suspected DVT (40). In contrast, the reported diagnostic yield for pelvic DVT in the setting of PFO and CS has varied between 1.5 and 13% (38, 39, 41). Of importance, DVT’s may develop due to the bed-ridden status of stroke patients therefore caution in interpreting this finding is warranted, particularly if discovered days after stroke onset. The suggestion of the role of PFO is CS has been postulated in cases of stroke with concomitant pulmonary embolism or myocardial infarction supporting a paradoxical embolic mechanism.

Beyond evaluation for DVT’s, pelvic MRV can help detect compression of the left iliac vein against the lumbar vertebra by the overlying right common iliac artery, known as the May–Thurner syndrome (42). Also known as iliocaval compression, the prevalence of this anatomical variant is roughly 20% and is more common among women (43). Although rarely symptomatic, May–Thurner syndrome as a plausible cause of paradoxical embolism in CS and PFO has a reported prevalence of 6.3% (44). Albeit an interesting finding, further studies are needed to evaluate the true role of May–Thurner syndrome in PFO-associated stroke.

Establishing the relationship between PFO and stroke is also challenging. A simple tool, the Risk of Paradoxical Embolism (ROPE) score has been proposed to risk stratify patients by age, and presence or absence of traditional vascular risk factors (45). Using clinical and radiological data of over 3000 patients, a multivariate regression model identified six variables associated with PFO status. A 10-point scale was created that can stratify the probability of a discovered PFO to be incidental or stroke related (Table 2). High ROPE scores are observed in younger patients with little or no traditional risk factors and superficially located infarcts, while low scores are observed in older patients with deep infarcts and traditional risk factors. In addition, an estimated 2-year risk of stroke or TIA was calculated for each group. Patients with higher probability of PFO-related stroke (ROPE scores 7–10) had the lowest recurrence rates (2–6%) while patients with lowest probability of PFO-related stroke (ROPE score 0–3) had the highest recurrence rate (20%) (Table 3) (45). These findings support the fact that patients with traditional stroke risk factors are more likely to have a PFO on their diagnostic workup as a “casual” relationship, rather than causal one.

**Table 2:** **Risk of paradoxical embolism score calculator (45)**.

Characteristic	Points
No history of hypertension	1
No history of diabetes	1
No history of stroke or TIA	1
Non-smoker	1
Cortical infarct on imaging	1
Age (years)	
18–29	5
30–39	4
40–49	3
50–59	2
60–69	1
≥70	0
Total score (sum of individual points)	
Maximum score (a patient <30 years with no hypertension, no diabetes, no history of stroke or TIA, non-smoker, and cortical infarct)	10
Minimum score (a patient ≥70 years with hypertension, diabetes prior stroke, current smoker, and no cortical infarct)	0

**Table 3:** **PFO prevalence, attributable fraction and estimated 2-year risk of stroke or TIA by points strata (using a control rate of 25%) (45)**.

	CS (*n* = 3023)			CS with PFO (*n* = 1324)
RoPE score	No. of patients	Prevelence of patients with a PF (95% CI)	PFO-attributable fraction,% (95% CI)	Estimated 2-year stroke/TIA recurrence rate, % (95% CI)
0–3	613	23 (19–26)	0 (0–4)	20 (12–28)
4	511	35 (31–39)	38 (25–48)	12 (6–18)
5	516	34 (30–38)	34 (21–45)	7 (3–11)
6	482	47 (42–51)	62 (21–45)	8 (4–12)
7	434	54 (49–59)	72 (66–76)	6 (2–10)
8	287	67 (62–73)	84 (79–87)	6 (2–10)
9	180	73 (66–79)	88 (83–91)	2 (0–4)

Patients with PFO, stroke and DVT should be anticoagulated, provided the risk of anticoagulation including bleeding and/or hemorrhagic transformation of an ischemic infarction does not outweigh the benefit of recurrent stroke. In such condition, and inferior vena cava filter may be considered (46). Endovascular management and reconstruction of occluded iliac veins in May–Thurner syndrome has low reported complication rates and is feasible in most cases (47). However, given the lack of randomized control trials in the setting of CS and PFO, careful consideration regarding optimal management is warranted, as true risk benefit has not been elucidated.

Current evidence-based guidelines do not support routine closure of PFO in the absence of DVT or proximal source and recommend antiplatelet therapy (46). Three major randomized controlled trials did not show a net benefit from closure of PFO in this population (48–50). A meta-analysis evaluating these 3 and an additional 11 non-randomized observational studies failed to prove superiority of closure against medical therapy, with an increased incidence of new onset AF in the closure group (RR 3.50) (51). However, it should be noted that previous PFO closure trials differed in study criteria, devices used, and lack of standardized design. Ongoing trials will hopefully find patients were ideal PFO closure is safe and with long-term data supporting decreased stroke risk and complications. At the present time, patients should be thoroughly evaluated and risk stratified before considering closure of PFO in ESUS.

Furthermore, current evidence-based guidelines do not recommend anticoagulation over antiplatelet therapy in patients with PFO (46). A large randomized control trial evaluating outcomes in PFO patients with anticoagulation vs. antiplatelet therapy with aspirin reported a 2-year event rates of 9.5% in the warfarin-treated group and 17.9% in the aspirin-treated group, but could not conclude statistical significance (HR, 0.5; 95% CI, 0.2–1.7). In addition, there was no significant difference between patients with isolated PFO, those associated with an atrial septal defect, or among small or large PFO’s (52). More recently, an individual participant meta-analysis evaluating oral anticoagulation or antiplatelet therapy in 2385 patients found no statistically significant difference in recurrent stroke, TIA, death; or stroke alone. Furthermore, subgroup analysis did not find significant heterogeneity of treatment effects in both groups, supporting the finding (53).

Considering a real-world experience of all treatment modalities, a recent analysis evaluated the net long-term benefit of different therapeutic strategies in patients with CS and PFO encompassing 3311 patients with ≥12 months of follow-up from both randomized and observational studies. Although anticoagulation therapy was more effective than antiplatelet therapy in preventing recurrent stroke and/or transient ischemic attack (event rates: 7.7 vs. 9.8%, respectively, *p* = 0.03), there was a sixfold greater risk of major bleeding (7.1 vs. 1.3%; odds ratio 6.49, 95% CI, 3.25–12.99, *P* < 0.00001). PFO closure was associated with 50% relative risk reduction of stroke and/or TIA vs. antiplatelet therapy and 82% relative reduction of major bleeding vs. anticoagulant therapy (54). Based on the current state, appropriate risk stratification, evaluation of individual stroke risk factors, and tailored therapy considering evidence-based guidelines are prudent.

#### Cardiac Imaging

Trans-thoracic echocardiography (TTE) and trans-esophageal echocardiography (TEE) are conventionally used to detect intra-cardiac sources of thrombi when investigating embolic stroke (55). TEE should be considered in patients with ESUS given its higher detection rate of valvular abnormalities including vegetation’s left atrial and ventricular thrombus, left atrial enlargement, and evaluation of the aortic arch irrespective of patients age (12). The utility of Cardiac MRI (CMRI) becomes important in detecting a cryptogenic source of stroke due to its overall greater sensitivity and high specificity in detecting LV thrombi, especially in the post MI sub-population (56, 57). CMRI is a multimodal technique utilizing a diverse subset of imaging such as spin echo, gradient echo as well as flow velocity encoding sequences giving enhanced resolution of cardiac anatomy (58). The utility of CMRI has been applied to gain enhanced imaging details such as fatty infiltration of the right ventricular free wall (causing cardiac rhythm abnormalities in right ventricle), evaluation of left and right ventricular cavity size and mass, as well as intra-cardiac shunts (59). In addition, a measure of blood flow is also obtained allowing the interpreter to objectify the severity of valvular regurgitation and stenosis (60). Through myocardial radio frequency tagging, a measure of myocardial dynamics can be obtained as well (61). The application of CMRI is especially valuable in areas pertaining to aortic arch disease (62) allowing enhanced visualization of dissection flaps, complex and false aortic aneurysms, atherosclerotic plaque, and supra-valvular aortic stenosis (63). Cine gradient echo CMR has also been used to visualize turbulence that is produced secondary to valvular stenosis/regurgitations (64, 65).

In an analysis of the utility of CMRI in etiology of CS, an evident or possible cardio-aortic source was found in 27.1% of patients. In addition, delayed enhancement-CMR (DE-CMR) sequences enabled further detection of potential sources (66). In this study, they were also able to show an overall higher sensitivity of CS detection via detection of an intra-cardiac source with CMR when compared to combination modalities such as TEE and TTE in detecting cardio-aortic as potential etiological origin (67). DE-CMR is also superior to conventional echocardiography in detecting transmural scarring, which constitutes an independent risk factor for LV mural thrombus when accompanied with LV wall motion abnormalities (68, 69). Despite advantages of CMR over conventional echocardiographic modalities, potential limitations decreasing its utility include cost of acquisition as well as relative paucity in availability (66, 70).

### Vascular Substenotic Atherosclerosis

Substenotic atherosclerotic plaques can possibly cause ischemic stroke by plaque rupture and artery-to-artery embolization. In particular, complicated atherosclerotic plaques with evidence of intraplaque hemorrhage on imaging have been suggested to be a potential mechanism in CS. Several modalities are currently used to detect complicated atherosclerotic plaques including high resolution magnetic resonance angiography (MRA). One study showed that complicated atherosclerotic carotid plaques with evidence of high intensity signal consistent with intraplaque hemorrhage in the internal carotid artery on MRA were present in approximately 25% of patients with CS and were more likely to occur ipsilateral vs. contralateral to the infarct (68). This finding was also confirmed by another study using the same imaging modality (71). These studies suggest that substenotic plaques with intraplaque hemorrhage may constitute a seperate mechanism in CS.

Furthermore, high resolution MRA with vessel wall imaging may be helpful in diagnosing intracranial atherosclerotic plaques. Diffuse enhancement of the vessel wall may be useful in diagnosing an active intracranial atherosclerotic plaque even in the absence of luminal narrowing, which may be a potential etiology in CS (12). The cost effectiveness of using advanced imaging modalities to look for complex, non-stenosing atherosclerotic plaques remains unclear. Moreover, their routine use remains controversial, especially since antiplatelet agents and statins are the mainstay of treating atherosclerotic-type strokes similar to treatments used in most patients with CS.

#### Aortic Arch Atherosclerosis

Aortic arch atherosclerosis (AAA) has been considered a risk factor for ESUS and CS. Complex plaque analysis during postmortem studies have demonstrated ulcerated aortic arch plaques covered with thrombi as well as cholesterol plaques as a nidus for embolism (72). Various studies demonstrated a direct correlation between AAA plaque size, characteristics, and stroke risk. Plaque size of 4 mm (RR of 4.7), mobile and/or complex plaque carry increased risk of embolism (46, 72). In particular, a dramatic rise in hazard ratio from 3.3 to 13.8 for AAA >4 mm in causing ESUS has been reported (73).

The Stroke Prevention in Atrial Fibrillation (SPAF) Echo trial, a meta-analysis of case–control case as well as postmortem series reported a significant odds of stroke in patients with severe AAA (3.76; 95% CI, 2.58–5.48) (74). In addition, AAA was found to be far more frequent and relatively higher in severity in patients >55 years. Additional systemic risk factors that influence AAA progression include cigarette smoking, diabetes, hypertension, and hypercholesterolemia (72). Vascular disease such as carotid atherosclerosis as well as peripheral vascular disease can serve as surrogate markers of AAA development risk due to an increased atheroma load (74).

Moreover, studies found a greater degree of embolic signals to the brain in patients with severe AAA compared to patients that did not (75–77). Atheroma from the mid-to-distal aortic arch has been reported to embolize to the left carotid artery territory. This has been suggested through studies using transcranial Doppler in identifying microembolic signals reaching the middle cerebral arteries compared to patients who did not have AAA-mediated emboli (75, 76).

Atherosclerotic plaques located in distal aortic arch also carry a potential risk for ESUS. A causative mechanism is amplified during diastole with retrograde blood flow from descending aorta reaching the major aortic branches in over 24% of patients with ESUS (78). While the relationship between AAA and ESUS has been reported in various trials, it is noteworthy to mention this relationship has been elucidated by only few studies with variable criteria in patient selection and definition of significant lesions (73, 76, 79–83). However, AAA should be considered as a cause of ESUS due to its relatively higher presence when compared to cervical artery atheroscelortic disease, especially after an extensive disease fails to demonstrate other arterial and cardiac causes (76, 84).

Thorough radiographic images of the aortic arch to investigate the extent of atherosclerotic changes as well as plaque characteristics such as thickness and degree of ulceration are needed. TEE, done primarily using two-dimensional as well as enhanced three-dimensional real time option give detailed representation of aortic plaque morphology and location (85). CT angiography (CTA) or MRI of the aortic arch may also be obtained. Besides providing a higher degree of resolution and being less invasive, additional details of the descending and abdominal aorta and its branches are visualized (86, 87). Conventional diagnostic catheter-based angiography is less applicable due to an overall less sensitivity for plaque detection and danger of plaque embolization during instrumentation (88).

In the PFO in Cryptogenic Stroke Study (PICSS), TEE detection of large plaques or complex morphology of aortic arch atheroma among patients with ESUS was associated with an overall increased risk of recurrent ischemic stroke or death over a 2-year follow up (HR, 6.42; 95% CI, 1.62–25.46) and (HR, 9.50; 95% CI, 1.92–47.10), respectively (89).

There is an overall paucity of conclusive data that suggests a net benefit of anticoagulation over anti-platelet therapy or vice versa. A retrospective study examining the effects of treatment on severe thoracic aortic plaques showed statin therapy (OR, 0.3; 95% CI, 0.2–0.6) but not warfarin (OR, 0.7; 95% CI, 0.4–1.2) or anti platelet therapy (OR, 1.4; 95% CI, 0.8–2.4) contributed toward decreasing risk of stroke, TIA, and peripheral embolization (90). Earlier studies suggested anticoagulation with warfarin decreased risk of recurrent stroke in patients with mobile thoracic atheroma (91) and benefit of anticoagulation over antiplatelet treatment in preventing recurrent strokes and peripheral embolic event in patients with aortic plaques >4 mm (92).

However, a prospective, randomized, controlled, open-labeled trial with blinded endpoint evaluation comparing the efficacy of aspirin plus clopidogrel to warfarin in patients with ischemic stroke, transient ischemic attack, or peripheral embolism with plaque in the thoracic aorta >4 mm, and no other identified embolic source was completed. Aspirin plus clopidogrel had a non-significant 24% reduction in the rate of recurrent stroke, myocardial infarction, peripheral embolism, and vascular death (adjusted *P* = 0.5) but significant reduction in vascular death compared with patients on warfarin (log-rank, *P* = 0.013). The results, while showing a marginal benefit of dual anti-platelet use over warfarin in preventing cerebral infarction, were not statistically significant rendering inconclusive results as the study was stopped prematurely (93).

Nevertheless, patients with aortic arch atheroembolization should be treated for secondary prevention with a combination of risk factor lowering strategies targeted at blood pressure, lipid control, and efficient glycemic control (94). Current guidelines recommend antiplatelet and statin therapy for patients with ischemic stroke or TIA and aortic arch atheroma, uncertainty of the effectiveness of anticoagulation in contrast to antiplatelet agents and no role for surgical endarterectomy (46).

#### Other Arterial Causes

Subtle luminal irregularities within the vascular distribution of an ischemic infarct should be carefully evaluated in ESUS and CS. Cervicocephalic large vessel dissections may account for up to a quarter of strokes in young adults under the age of 45 years (95). Of importance, the majority of dissections are spontaneous therefore lack of trauma history should not rule out dissection as a possible etiology. MRA with T1 fat saturation can show intramural blood and expansion and therefore should be considered in patients with suspected dissection (96).

Varicella Zoster Virus (VZV)-related vasculopathy has a broad clinical spectrum of conditions including TIA, ischemic strokes, hemorrhagic infarcts, aneurysm formation, and intra-parenchymal and subarachnoid hemorrhage due to both large and small vessel vasculitis (97). Approximately 40% of transient cerebral arteriopathy and a third of ischemic stroke of arterial origin in the pediatric population are attributed to VZV (98, 99). In addition, up to a third of adults who had VZV infection may suffer a stroke within 1 year of infection (100, 101). Large vessel unifocal vasculopathy is more commonly observed with zoster-ophthalmicus, while both large and small vessel vasculopathy is seen win immunocompromised patients (102). In a study of 30 patients with VZV vasculitis, 97% had abnormal brain MRI with the majority of ischemic changes involving the deep structures such as basal ganglia (102). Only 63% of patients manifested a skin rash, and angiographic evidence of arterial stenosis was present in 70% of the cases (37% only small vessels, 13% only large vessels, and 50% had mixed presentation) (102).

In patients with CS, history of VZV infection (particularly in the ophthalmic distribution) or immune-compromised state should prompt evaluation. Serologic testing of CSF should include VZV IgG (93% sensitive) as DNA-PCR sensitivity is low (30% sensitive) (102). Diagnosed cases of VZV vasculopathy should be treated with 10–15 mg/kg acyclovir for a minimum of 14 days (97). Adjunct steroid treatment (1 mg/kg Prednisone) can be considered without taper for 5 days but not to be extended beyond 1 week due to possibility of potentiation of viral infection. In cases not responsive to acyclovir therapy, prolonged valacyclovir 1gm TID dosing for 1–2 months may be considered (97). Other infectious causes for stroke such as neuroborrelosis or syphilis are rare but should also be considered in patients with CS or ESUS based on history and clinical findings.

### Hypercoagulable States

Hypercoagulable states have a reported prevalence of 3–21% in ischemic stroke (103). Inherited coagulopathies most commonly invoke venous thrombosis (104, 105). Antiphospholipid antibodies and homocystienemia involve both arterial and venous systems as compared to other pro-coagulant states. In patients with idiopathic or recurrent thrombophilia manifesting in cerebral ischemic events, a diagnostic work-up is warranted. The different mechanisms contributing to a hypercoaguable state with corresponding testing in a hierarchical manner corresponding to the degree of evidence is depicted in Table 4.

**Table 4:** **Implicated hypercoagulable states in stroke (103, 104, 10,4)**.

	Prevalence	Probable	Possible	Equivocal
**Inherited hypercoagulable states**				
Coagulation proteins defects	0–21%	Fibrinogen level, prothrombin G20210A variant factor V Leiden, protein C, protein S, Antithrombin III	Factor VIII level	Factor XII, Factor XIII, vWF Smal polymorphism in intron 2
Fibrinolytic system defects	0–2.7%		Plasminogen activator inhibitor type 1	Tissue plasminogen activator	
Platelets hyper-reactivity	No evidence	–	–	–
Biochemical	Difficult to identify direct contribution due to multifactorial process	Hyperhomocysteinemia, MTHFR c677t	C-reactive protein, lipoprotein a, paraoxonase 1, endothelial nitric oxide synthase, apo-lipoprotein, transforming growth factor B1, P-selectin angiotensin converting enzyme, P-selectin	
**Acquired hypercoagulable states**				
	Not enough data based on studies	Oral contraceptive pills, hormone replacement therapy, history of idiopathic venous thrombosis, malignancy	Thrombotic thrombocytopenic purpura, heparin-induced thrombocytopenia	
**Others**				
	5–20%	Antiphospholipid syndrome		
	5–10%	Myeloproliferative disorders		
	Sickle cell anemia (15%), Sickle-C disease (2–5%), Sickle cell trait (1.5–2%)	Sickle cell disease		

The prevalence of stroke related to polygenic and acquired risk factors is accelerated with age, albeit this association is reversed in monogenetic mutations (104). Typically, age <60, history of minimal traditional vascular risk factors, recurrent unprovoked venous and arterial thrombotic events, and positive family history in a patient profile would increase the yield for a positive test (103, 104, 106). Furthermore, measuring homeostatic factors at the time of hospitalization can be of less diagnostic yield owing to the derangement of the otherwise normal metabolic profile of a patient in the setting of acute illness (i.e., thrombotic state, hepatic disease, renal disease, traumatic-acute stress, sepsis, malignancy, malnutrition, pregnancy, anticoagulation medication use) (107). In addition to clinical criteria, positive antiphospholipid antibodies (lupus anticoagulant, anticardiolipin, or anti-beta-2-glycoprotien) require repeated testing 12 weeks later to confirm antiphopsholipid syndrome (108). Testing of Factor V leiden mutation and Prothrombin gene mutation can be performed immediately during hospitalization; however, measuring levels of protein C, protein S, or antithrombin deficiency should be deferred 2–3 month after hospitalization or when patients are not on anticoagulation (109, 110). Despite limited evidence of prophylactic therapy, once the diagnosis is confirmed anticoagulation for inherited thrombophilia may be considered as directed by current guidelines and recommendations (American Heart Association-Level IIa, Class C) (111).

### Stroke Related to Cancer

Ischemic stroke incidence can be as high as 15% in patients with malignancy, of which only 50% are identified (112). Trousseau syndrome refers to spontaneous recurrent or migratory episodes of arterial emboli due to non-bacterial thrombotic endocarditis, venous thrombosis, or both in a patient with an underlying malignant neoplasm. Several biochemical processes related to the hypercoagulable state of malignancy have been suggested. These include change in the homeostatic property of blood and blood vessels that involve activation of cell adhesion molecules by mucin secreted from adenocarcinomas (113, 114), release of tissue factors by cancer cells causing activation of factor VII and X (115–118), endothelial cell damage from procoagulant cytokines such as TNF-alpha, IL-1, and IL-6, which cause vWF release (117, 118), platelet activation, protein C inhibition (119), intravascular lymphomatosis (120), and increased viscosity observed in myeloproliferative disorders (121).

Occult cancer as a cause of CS should be considered once traditional risk factors have been ruled in patients with suggestive clinical history, advanced age, or familial cancers. In a study comparing MRI findings of stroke in cancer patients with the general population, the rate of multiple territory infarcts was fourfold higher. In addition, gastrointestinal cancer had strong correlation with embolic strokes (122). Perhaps multiple territory infarctions may serve as a potential radiographic marker of underlying malignancy in high-risk individuals with ESUS or CS. Owing to the heterogeneity of malignancy subtypes, there is currently no standardized approach to screening. However, based on individual patient profiles and contributing risk factors, cancer screening should be individualized. Age appropriate cancer screening modalities, D-dimer, sedimentation rate, C-reactive protein, and CT scan of Chest, Abdomen, and Pelvis are some of the most commonly used techniques for the malignancy workup (123).

While recognizing that there is limited evidence for stroke prevention in cancer-related hypercoagulability states, it becomes imperative to establish a rapid diagnosis of the underlying malignancy to intimate appropriate therapy. Anticoagulation therapy in patients with malignancy-related hypercoagulable state is effective and decreases D-dimer levels over time; a surrogate marker for hypercoagulability in malignancy (124). The use of antiplatelet agents instead of anticoagulation if the pattern of strokes resembles small vessel related infarcts has also been documented (123). However, prophylaxis of venous thromboembolic events in cancer patients with low-molecular Heparin (LMWH) has some reported degree of superiority of over Warfarin (123, 125).

### Other Potential Causes

#### Migraine

A pooled analysis of studies analyzing the correlation between migraines and stroke demonstrated a RR of 2.04 (95% CI, 1.72–2.43) (126) with an exacerbated risk in the migraine with aura subtype. Gender correlations in women who experience migraines have shown an overall higher risk of strokes than men (127). Nevertheless, a relationship between men and the elderly who experience migraines and strokes also exists (128). Other recognized risk factors in patients with migraines causing an increased risk of stroke include frequency of attacks (more than weekly), absence of nausea/vomiting, age >45 years, history of smoking, and oral contraceptive use (127, 129–131). In the Oxford Vascular Study, 37% of strokes and TIA’s in a CS population (over 1000 patients) had associated migraines (with or without auras) (132). Moreover, a strong link between patients older than 65 years of age with fewer vascular risk factors, no family history of stroke, and elevated frequency of migraines to have a high degree of ESUS (132). Potential mechanisms explaining the link between migraines and strokes have spanned from the presence of anatomical variants of circle of Willis, basilar hypoplasia, infratentorial lacunar lesions (133) to disturbances in the cortical excitability, cortical depression, and trigeminovascular system activation (134). Other potential risk factors highlighted in literature as a causative link between migraines and strokes include hypercoaguable states, hypoperfusion, and PFO’s. The relationships between PFO’s and stroke in migraine have been looked upon extensively (35, 135, 136). One potential mechanism is thought to be related to paradoxical emboli across the PFO going ultimately to the brain, also triggering migraines (137). However, trials for PFO closure as a potential source of decreasing migraines showed an overall lack of such an association (138–140).

Infarcts in patients with migraine have a predilection for the posterior circulation, and most commonly are silent, small, multiple, located within the vascular borderzone location in the cerebellum, and more prevalent in patients with aura (141). A migrainous infarction is defined as one or more migrainous aura symptoms typical for a previous attack lasting >60 min in a patient with migraine with aura, associated with an ischemic brain lesion in appropriate territory demonstrated by neuroimaging not attributed to another disorder (142). Migrainous infarction incidence represents 0.5–1.5% of all ischemic stroke but up to 10–14% in of ischemic stroke in the young (143). Recognition of such infarcts is important particularly in women with frequent migraine with aura, posterior circulation territory location and no identifiable cause after thorough investigation.

The Womens Health study highlighted the beneficial role of aspirin in reducing the risk of ischemic stroke in patients with migraines (RR 0.76; 95% CI, 0.63–0.93). This benefit was especially seen in women more than 45 years of age and aspirin should be considered in these patients. Due to reports highlighting the relationship between PFO’s, migraines, and ESUS, the Migraine Intervention with STARFlex Technology (MIST) trial was completed but failed to show any benefit of PFO closure on cessation of migraine headaches (138). In addition, various trials as well as a recent meta-analysis failed to show a conclusive association between PFO and migraines (140, 144, 145). Thus far, data is quite limited on evidence of migraine prophylaxis and reduction of stroke risk (146). Ultimately practice guidelines in primary prevention of stroke in patients with migraines have thus stressed smoking cessation in women with migraines of the aura subtype (WMA), avoidance of oral contraceptive agents with estrogen in WMA, treating to reduce migraine frequency, and no clear benefit of PFO closure as a means to prevent strokes in patients with migraines (146). It should be noted, however, that the epidemiological studies examining PFO closure, migraines, and CSs predominantly involved elderly population and failed to include younger patients more exclusively. Therefore, for now adequate management of hypertension, hyperlipidemia, and diabetes mellitus in patients with migraines is recommended by the American Headache Society (128, 147–151). In addition, patients suffering from cardiovascular disease, hemiplegic, or basilar migraines should also avoid vasoconstrictive drugs such as triptans and ergotamines (128, 147–151).

#### Fabry Disease

Fabry disease and inherited progressive X-linked disorder of glycosphingolipid metabolism due to alpha-galactosidase A (alpha-Gal A) deficiency represent approximately 1% of all stroke in the young but between 3 and 5% of CS (152). Patients typically are younger males and exhibit a constellation of symptoms including neuropathy, angiokeratomas, cardiovascular, renal, and neurological manifestations (153–156). Ischemic strokes in Fabry disease have a predilection to the posterior circulation and are associated with vertebrobasilar dolichoectasia (157). Vasculopathy and arteriopathy in Fabry disease may manifest as TIA’s, strokes, aneurysms, and occular symptoms such as blindness (158–163). Ischemic stroke is the predominant subtype (5.6%) with mean age <30 and <45 in men and women, respectively (164, 165). Serum measurement of leukocyte alpha-Gal A activity is standard in most laboratories as work up for Fabry’s disease. However, the sensitivity of the test varies in males and females; being almost 100% in males but positive in only 50% of female carriers and hemizygous males (166, 167). Antiplatelet therapy, enzyme replacement therapy (ERT), and risk factor modification are the mainstay secondary preventative measures of treatment in Fabry disease. Kidney transplantation may be considered in very advanced cases with complications arising from CKD (168–171).

#### Homocysteine

The relationship between hyperhomocysteinemia (HHC) and ESUS remains relatively underexplored, and often with contradictory results (172–174). In a study examining the relationship between HHC and stroke, patients with CS had moderately elevated levels of homocysteine when compared to controls. However, this effect was nullified when cardiovascular risk factors were adjusted (175). In other studies, a neutral association of HHC with CS has also been reported (172, 176). Some suggest HHC serves as a marker for atherosclerosis, as well as other cardiovascular risk factors leading to stroke (172). A relationship of HHC and CS is, however, amplified in the presence of obesity (175, 177–179). This observation is potentially explained due to increased oxidative stress parameters and obesity-related hormones, which would amplify HHC as a causative agent (175).

Elevated levels of homocysteine can be attributed to multiple causes. Genetic defects in cystathionine beta synthase and methylenetetrahydrofolate reductase (MTHFR) can result in homocysteine level elevation. Decreased renal clearance, especially in patients with chronic kidney disease can also contribute toward elevated HC levels (180). In addition, nutritional deficiencies encompassing Vitamin B6, Vitamin B12, and Vitamin B9 (folate) can also cause elevations in HC (181–183).

Conflicting data exist on treatment of elevated homocysteine levels by vitamin supplementation. A meta-analysis of 26 randomized controlled trials that found a trend toward decreased stroke risk (RR 0.93; 95% CI, 0.86–1.00) in patients supplementing with folic acid (184). Similarly, in the HOPE 2 study, a combination of Vitamin B6, B12, and Folic acid did lower plasma HC levels and reduced stroke risk by 25% (RR 0.75; 95% CI, 0.59–0.97) (185). However, despite the somewhat positive results of the aforementioned trials regarding vitamin supplementation and stroke risk, the overall bulk of trials thus far examining the effect of Vitamin B complex vitamin and risk of stroke are inconsistent. Nevertheless, treatment of HHC with Vitamin B and folate supplementation strategies to reduce overall ischemic stroke risk should be considered, but with an overall undetermined efficacy (146).

#### Other Genetic, Autoimmune, or Rheumatologic Causes

Although the reported prevalence of an underlying genetic abnormality as the cause of ischemic stroke is quite low (<1%), early identification of a genetic abnormality may have relevant implications for management and future counseling (186). More importantly, the true prevalence in ESUS or CS is unknown. For patients with stroke at a young age, positive family history, and absence of conventional risk factors, there is higher probability of a genetic cause and yield of testing (187). It may be prudent to consider genetic entities (e.g., monogenic vessel diseases) in selected individuals.

One should consider Susac syndromes when ocular manifestations predominant and occur on a recurrent basis (188, 189). The typical triad of this autoimmune endotheliopathy consists of encephalopathy, branch retinal artery occlusion and hearing loss (190). The disease manifests more in young women (22–42) and the age range overall can affect patients from 7 to 72 years of age (190). Strokes commonly involve the corpus collosom.

Up to 20% of SLE patients suffer from stroke with further studies elucidating its relationship after controlling for age and gender with an odds ratio of 1.5 (191–193). Antiphospholipid antibody (APa) syndrome tends to be the most frequent condition associated with arterial hypercoagulability manifesting as thrombocytopenia, livedo reticularis (Sneddons syndrome), pre-eclmapsia, and still birth (190). Additional mechanisms of strokes can be secondary to accelerated atherosclerosis, Libman–Sacks endocarditis, elevated homocysteine, and vasculitic mechanisms (194–196). Other autoimmune and rheumatologic conditions affecting the intracranial and/or extracranial vasculature (e.g., Giant cell, Takayasu’s, Polyarteritis nodosa, Kawasaki’s, microscopic polyangiitis, Eosinophilic granulomatosis, Behcets’s, Cogan’s syndrome, granulomatosis with polyangiits, Sarcoidosis, etc.) are beyond the scope of this chapter and discussed elsewhere (197). However, these should be considered in the evaluation of patients with CS with subtle luminal irregularities and suggestive symptoms, clinical features, or positive serology during evaluation.

#### Diagnostic Work-Up

The first step in evaluation of a patient with ESUS would be a thorough physical exam including evaluation of the skin and careful review of personal and family history. Appropriate classification is mandatory as the yield; cost and extent of the diagnostic work-up will vary. In the absence of conventional risk factors and in patients with ESUS, ancillary testing such as use of TEE, CMRI is required for cardiac imaging. Prolonged cardiac monitoring should be considered in all patients with ESUS. Patients with a PFO should have lower extremity ultrasonography and/or MRV of the pelvis. Sub-stenotic arterial lesions require careful evaluation including MRA, T1 fat saturation (if dissection is suspected), or conventional catheter angiography if subtle or multifocal irregularities are found, particularly in younger patients.

**Figure 1:**
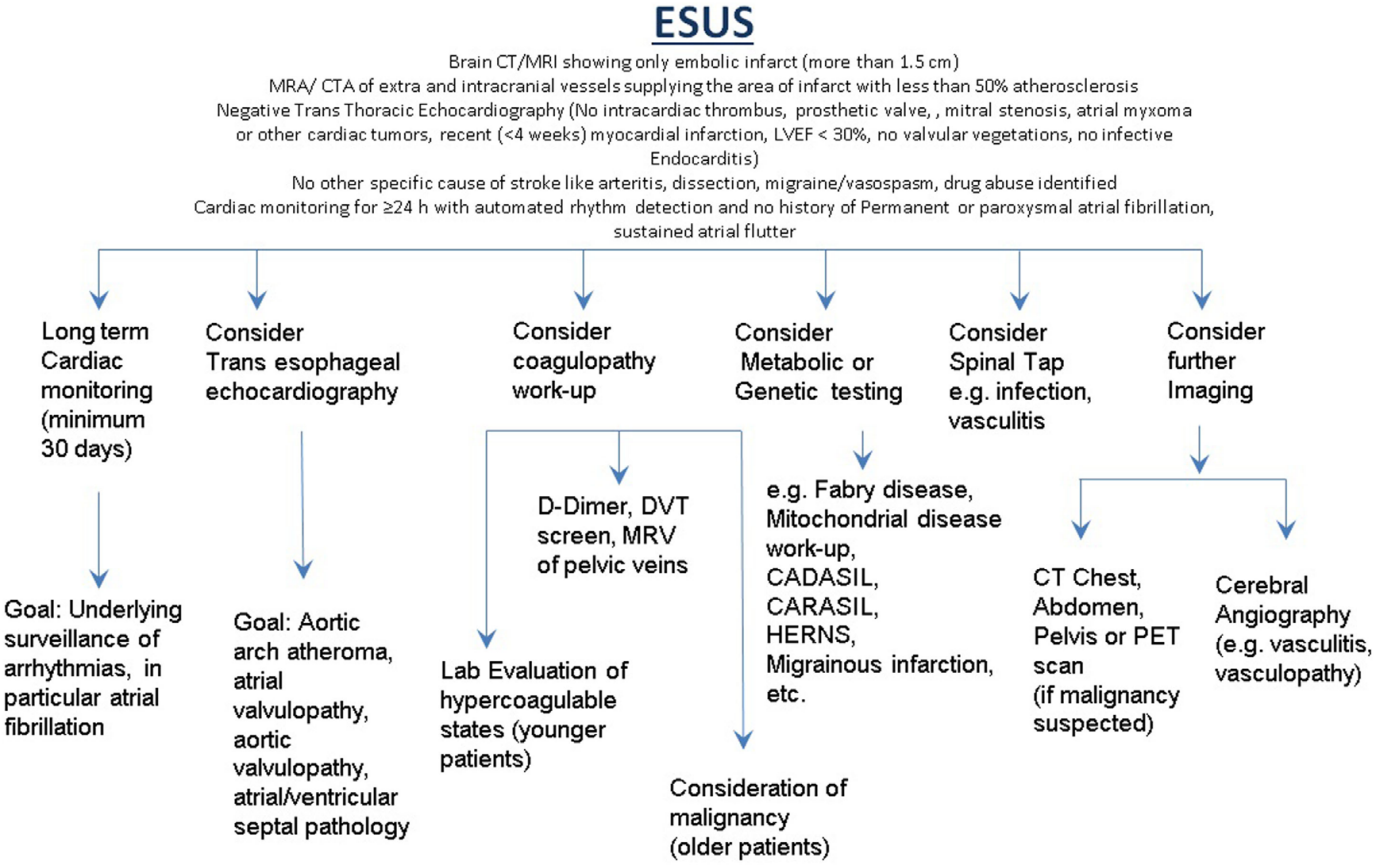
**Suggested investigative approach for patients with ESUS**.

Patients with associated systemic manifestations suggesting infectious or inflammatory conditions may require CSF examination. Appropriate screening for hypercoagulable state in younger patients with recurrent thrombosis or family history may be appropriate. Multi-vessel territory infracts in older patients with suggestive individual profiles or clinical history may require investigation for malignancy. A suggested investigative approach for patients with ESUS is illustrated in Figure 1.

#### Management

As stroke is >80% preventable, patients with any conventional stroke risk factors must be adequately managed to prevent recurrence. Treatments pertaining to the specific conditions in this article have been discussed. However, antiplatelet therapy remains the mainstay for antithrombosis in ESUS and CS at the present time. Two randomized clinical trials are currently investigating the use of novel oral anticoagulants in ESUS patients as compared to antiplatelet therapy. The RE-SPECT ESUS trial compares dabigatran 150 or 100 mg twice daily to aspirin 100 mg daily for secondary prevention in a double-blinded fashion with a follow-up period of up to 3 years (198). Similarly, the NAVIGATE ESUS trial will compare rivaroxaban 15 mg daily to aspirin 100 mg daily for secondary prevention in patients with ESUS (199). These trials and similar future trials are needed to establish the best treatment modality for patients with ESUS and non-ESUS CS stroke patients.

#### Future Directions and Conclusion

A novel and relatively underexplored area of research into identifying stroke etiology has been analysis of clots (thrombus) mechanically retrieved after endovascular therapy for stroke. Understanding thus far has been limited due to the relative novelty of these procedures and an overall lack of standardization or supporting clinical trials, until recently.

Marder et al. (200) presented one of the first clot analysis studies in 2005 and indicated that histologic analysis of clots retrieved from large intracerebral vessels might point toward the etiology of stroke. The study could not reach to a statistically significant conclusion due to low sample size. The results were also affected as the study was done using MERCI device (an older generation device), which disrupts clot architecture significantly. Since 2005, newer generation devices and corresponding clinical trials have shown with enough evidence, the efficacy of these devices in successful clot retrieval. A recently published study demonstrated that higher red cell content in the clot retrieved from acute interventional stroke treatment showed correlation with a cardioembolic source. Although promising, this study due to the low numbers of patients analyzed could not reach a statistical conclusion (201). Further histopathological studies are needed to help establish standardized clot characteristics correlating the embolic source. In turn, this may be a new diagnostic avenue in ESUS and CS.

Biomarkers such as CK-MB and BNP have been suggested to improve the diagnosis of embolic ischemic stroke in ESUS. In one study, high levels of both markers were predictors of acute phase embolism in cardioembolic strokes with combined specificity of 95% and PPV of 88.2% (202). Increased visceral adipose tissue has been recently identified as a potential risk factor of stroke in ESUS. Increased VAT was found in over half of patients with ESUS in on study and was more common than any other conventional stroke risk factor (203). More recently, PET scanning with a fibrin-binding probe tracer injected after thrombosis has been shown to successfully detect multiple thrombi both venous and arterial in rats, with accurate composition. Probe uptake was greater in younger vs. older clots (204). Perhaps a future role for such technology would include stroke patients, investigating the proximal source of a distal thrombus soon after ischemic stroke.

These future investigations may help establish the true source of embolism and therefore appropriately dictate treatment. However, for patients with truly CS further clinical trials are needed for optimal management and secondary prevention.

## Author Contributions

This review article was lead by AN, with secondary contributions from MH, TM, and SY. A thorough review of the literature was completed by AN, with contributions from all the other authors. AN completed the introduction, abstract, discussion, and parts of the cardiac section and oversaw the entire article write up. In addition, AN completed the review of the contributions from other authors. Edits including text correction, grammar, and stylistic review was completed by AN. The final review was completed after all contributions from all authors was completed. All tables and diagrams were reviewed by all authors.

## Conflict of Interest Statement

Dr. SY received funding from the New York Stroke Trials Network of Columbia and Cornell (NYCCSTN, NINDS U10NS086728). The remaining authors declare that the research was conducted in the absence of any commercial or financial relationships that could be construed as a potential conflict of interest.

## References

[1] Grau AJ Weimar C Buggle F Heinrich A Goertler M Neumaier S Risk factors, outcome, and treatment in subtypes of ischemic stroke: the German stroke data bank. Stroke (2001) 32(11):2559–66. 10.1161/hs1101.098524 11692017

[2] Putaala J Metso AJ Metso TM Konkola N Kraemer Y Haapaniemi E Analysis of 1008 consecutive patients aged 15 to 49 with first-ever ischemic stroke the Helsinki young stroke registry. Stroke (2009) 40(4):1195–203. 10.1161/STROKEAHA.108.529883 19246709

[3] Li L Yiin GS Geraghty OC Schulz UG Kuker W Mehta Z Incidence, outcome, risk factors, and long-term prognosis of cryptogenic transient ischaemic attack and ischaemic stroke: a population-based study. The Lancet Neurology (2015) 14(9):903–13. 10.1016/S1474-4422(15)00132-5 26227434 PMC5714616

[4] Adams HP Jr Bendixen BH Kappelle LJ Biller J Love BB Gordon DL Classification of subtype of acute ischemic stroke. Definitions for use in a multicenter clinical trial. TOAST. Trial of Org 10172 in Acute Stroke Treatment. Stroke 24 (1993) 1:35–41. 10.1161/01.STR.24.1.35 7678184

[5] Amarenco P Bogousslavsky J Caplan LR Donnan GA Hennerici MG New approach to stroke subtyping: the ASCO (phenotypic) classification of stroke. Cerebrovasc Dis (2009) 27(5):502–8. 10.1159/000210433 19342826

[6] Ji R Schwamm LH Pervez MA Singhal AB. Ischemic stroke and transient ischemic attack in young adults: risk factors, diagnostic yield, neuroimaging, and thrombolysis. JAMA Neurol (2013) 70(1):51–7. 10.1001/jamaneurol.2013.575 23108720

[7] Hart RG Diener HC Coutts SB Easton JD Granger CB O’Donnell MJ Embolic strokes of undetermined source: the case for a new clinical construct. Lancet Neurol (2014) 13(4):429–38. 10.1016/S1474-4422(13)70310-7 24646875

[8] Ntaios G Papavasileiou V Milionis H Makaritsis K Vemmou A Koroboki E Embolic strokes of undetermined source in the Athens stroke registry: an outcome analysis. Stroke (2015) 46(8):2087–93. 10.1161/STROKEAHA.115.009334 26159795

[9] Goldstein LB Bushnell CD Adams RJ Appel LJ Braun LT Chaturvedi S Guidelines for the primary prevention of stroke: a guideline for healthcare professionals from the American Heart Association/American Stroke Association. Stroke (2011) 42:517–84. 10.1161/STR.0b013e3181fcb238 21127304

[10] Gladstone DJ Spring M Dorian P Panzov V Thorpe KE Hall J Atrial fibrillation in patients with cryptogenic stroke. N Engl J Med (2014) 370:2467–77. 10.1056/NEJMoa1311376 24963566

[11] Sanna T Diener HC Passman RS Di Lazzaro V Bernstein RA Morillo CA Cryptogenic stroke and underlying atrial fibrillation. N Engl J Med (2014) 370:2478–86. 10.1056/NEJMoa1313600 24963567

[12] Yaghi S Elkind MS. Cryptogenic stroke: a diagnostic challenge. Neurol Clin Pract (2014) 4:386–93. 10.1212/CPJ.0000000000000086 25317376 PMC4196459

[13] Favilla CG Ingala E Jara J Fessler E Cucchiara B Messe SR Predictors of finding occult atrial fibrillation after cryptogenic stroke. Stroke (2015) 46:1210–5. 10.1161/STROKEAHA.114.007763 25851771

[14] Bernstein RA Di Lazzaro V Rymer MM Passman RS Brachmann J Morillo CA Infarct topography and detection of atrial fibrillation in cryptogenic stroke: results from CRYSTAL AF. Cerebrovasc Dis (2015) 40:91–6. 10.1159/000437018 26182860

[15] Miller DJ Khan MA Schultz LR Simpson JR Katramados AM Russman AN Outpatient cardiac telemetry detects a high rate of atrial fibrillation in cryptogenic stroke. J Neurol Sci (2013) 324:57–61. 10.1016/j.jns.2012.10.001 23102659

[16] Gladstone DJ Dorian P Spring M Panzov V Mamdani M Healey JS Atrial premature beats predict atrial fibrillation in cryptogenic stroke: results from the embrace trial. Stroke (2015) 46:936–41. 10.1161/STROKEAHA.115.008714 25700289

[17] Glotzer TV Daoud EG Wyse DG Singer DE Ezekowitz MD Hilker C The relationship between daily atrial tachyarrhythmia burden from implantable device diagnostics and stroke risk: the trends study. Circ Arrhythm Electrophysiol (2009) 2:474–80. 10.1161/CIRCEP.109.849638 19843914

[18] Brambatti M Connolly SJ Gold MR Morillo CA Capucci A Muto C Temporal relationship between subclinical atrial fibrillation and embolic events. Circulation (2014) 129:2094–9. 10.1161/CIRCULATIONAHA.113.007825 24633881

[19] Keach JW Bradley SM Turakhia MP Maddox TM. Early detection of occult atrial fibrillation and stroke prevention. Heart (2015) 101:1097–102. 10.1136/heartjnl-2015-307588 25935765

[20] Kamel H Elkind MS Bhave PD Navi BB Okin PM Iadecola C Paroxysmal supraventricular tachycardia and the risk of ischemic stroke. Stroke (2013) 44:1550–4. 10.1161/STROKEAHA.113.001118 23632982 PMC3950597

[21] Yaghi S Moon YP Mora-McLaughlin C Willey JZ Cheung K Di Tullio MR Left atrial enlargement and stroke recurrence: the Northern Manhattan Stroke Study. Stroke (2015) 46:1488–93. 10.1161/STROKEAHA.115.008711 25908460 PMC4442058

[22] Kamel H O’Neal WT Okin PM Loehr LR Alonso A Soliman EZ Electrocardiographic left atrial abnormality and stroke subtype in atherosclerosis risk in communities study. Ann Neurol (2015) 78(5):670–8. 10.1002/ana.24482 26179566 PMC4624007

[23] Kamel H Soliman EZ Heckbert SR Kronmal RA Longstreth WT Jr Nazarian S P-wave morphology and the risk of incident ischemic stroke in the multi-ethnic study of atherosclerosis. Stroke (2014) 45:2786–8. 10.1161/STROKEAHA.114.006364 25052322 PMC4146624

[24] Sinner MF Stepas KA Moser CB Krijthe BP Aspelund T Sotoodehnia N B-type natriuretic peptide and c-reactive protein in the prediction of atrial fibrillation risk: the CHARGE-AF consortium of community-based cohort studies. Europace (2014) 16:1426–33. 10.1093/europace/euu175 25037055 PMC4197895

[25] Folsom AR Nambi V Bell EJ Oluleye OW Gottesman RF Lutsey PL Troponin t, n-terminal pro-b-type natriuretic peptide, and incidence of stroke: the atherosclerosis risk in communities study. Stroke (2013) 44:961–7. 10.1161/STROKEAHA.111.000173 23471272 PMC3614093

[26] Cushman M Judd SE Howard VJ Kissela B Gutierrez OM Jenny NS N-terminal pro-b-type natriuretic peptide and stroke risk: the reasons for geographic and racial differences in stroke cohort. Stroke (2014) 45:1646–50. 10.1161/STROKEAHA.114.004712 24757103 PMC4142424

[27] Yaghi S Boehme AK Hazan R Hod EA Canaan A Andrews HF Atrial cardiopathy and cryptogenic stroke: a cross-sectional pilot study. J Stroke Cerebrovasc Dis (2015) 25(1):110–4. 10.1016/j.jstrokecerebrovasdis.2015.09.001 26476588 PMC4695267

[28] Longstreth WT Jr Kronmal RA Thompson JL Christenson RH Levine SR Gross R Amino terminal pro-b-type natriuretic peptide, secondary stroke prevention, and choice of antithrombotic therapy. Stroke (2013) 44:714–9. 10.1161/STROKEAHA.112.675942 23339958 PMC3583375

[29] Homma S Sacco RL Patent foramen ovale and stroke. Circulation (2005) 112:1063–72. 10.1161/CIRCULATIONAHA.104.524371 16103257

[30] Meier B Frank B Wahl A Diener HC. Secondary stroke prevention: patent foramen ovale, aortic plaque, and carotid stenosis. Eur Heart J (2012) 33(6):705–13. 10.1093/eurheartj/ehr443 22422912 PMC3303713

[31] Alsheikh-Ali AA Thaler DE Kent DM. Patent foramen ovale in cryptogenic stroke incidental or pathogenic? Stroke (2009) 40(7):2349–55. 10.1161/STROKEAHA.109.547828 19443800 PMC2764355

[32] Lethen H Flachskampf FA Schneider R Sliwka U Köhn G Noth J Frequency of deep vein thrombosis in patients with patent foramen ovale and ischemic stroke or transient ischemic attack. Am J Cardiol (1997) 80(8):1066–9. 10.1016/S0002-9149(97)00604-8 9352979

[33] Handke M Harloff A Olschewski M Hetzel A Geibel A. Patent foramen ovale and cryptogenic stroke in older patients. N Eng J Med (2007) 357(22):2262–8. 10.1056/NEJMoa071422 18046029

[34] Wessler BS Thaler DE Ruthazer R Weimar C Di Tullio MR Elkind MS Transesophageal echocardiography in cryptogenic stroke and patent foramen ovale analysis of putative high-risk features from the risk of paradoxical embolism database. Circ Cardiovasc Imaging (2014) 7(1):125–31. 10.1161/CIRCIMAGING.113.000807 24214884 PMC3934652

[35] Overell JR Bone I Lees KR. Interatrial septal abnormalities and stroke: a meta-analysis of case-control studies. Neurology (2000) 55(8):1172–9. 10.1212/WNL.55.8.1172 11071496

[36] Messe SR Silverman IE Kizer JR Homma S Zahn C Gronseth G Practice parameter: recurrent stroke with patent foramen ovale and atrial septal aneurysm report of the Quality Standards Subcommittee of the American Academy of Neurology. Neurology (2004) 62(7):1042–50. 10.1212/01.WNL.0000119173.15878.F3 15078999

[37] Claver E Larrousse E Bernal E López-Ayerbe J Valle V. Giant thrombus trapped in foramen ovale with pulmonary embolus and stroke. J Am Soc Echocardiogr (2004) 17(8):916–8. 10.1016/j.echo.2004.04.020 15282501

[38] Liberman AL Daruwalla VJ Collins JD Maas MB Botelho MPF Ayache JB Diagnostic yield of pelvic magnetic resonance venography in patients with cryptogenic stroke and patent foramen ovale. Stroke (2014) 45(8):2324–9. 10.1161/STROKEAHA.114.005539 24938843

[39] Osgood M Budman E Carandang R Goddeau RP Jr Henninger N. Prevalence of pelvic vein pathology in patients with cryptogenic stroke and patent foramen ovale undergoing MRV pelvis. Cerebrovasc Dis (2015) 39(3–4):216–23. 10.1159/000376613 25791718

[40] Spritzer CE Arata MA Freed KS Isolated pelvic deep venous thrombosis: relative frequency as detected with MR imaging 1. Radiology (2001) 219(2):521–5. 10.1148/radiology.219.2.r01ma25521 11323482

[41] Cramer SC Rordorf G Maki JH Kramer LA Grotta JC Burgin WS Increased pelvic vein thrombi in cryptogenic stroke results of the Paradoxical Emboli From Large Veins in Ischemic Stroke (PELVIS) Study. Stroke (2004) 35(1):46–50. 10.1161/01.STR.0000106137.42649.AB 14657451

[42] McDermott S Oliveira G Ergül E Brazeau N Wicky S Oklu R May-Thurner syndrome: can it be diagnosed by a single MR venography study. Diagn Interv Radiol (2013) 19(1):44–8. 10.4261/1305-3825.DIR.5939-12.1 22801870

[43] May R Thurner J The cause of the predominantly sinistral occurrence of thrombosis of the pelvic veins. Angiology (1957) 8(5):419–27. 10.1177/000331975700800505 13478912

[44] Kiernan TJ Yan BP Cubeddu RJ Rengifo-Moreno P Gupta V Inglessis I May-Thurner syndrome in patients with cryptogenic stroke and patent foramen ovale: an important clinical association. Stroke (2009) 40(4):1502–4. 10.1161/STROKEAHA.108.527366 19182088 PMC3744111

[45] Kent DM Ruthazer R Weimar C Mas JL Serena J Homma S An index to identify stroke-related vs incidental patent foramen ovale in cryptogenic stroke. Neurology (2013) 81(7):619–25. 10.1212/WNL.0b013e3182a08d59 23864310 PMC3775694

[46] Kernan WN Ovbiagele B Black HR Bravata DM Chimowitz MI Ezekowitz MD Guidelines for the prevention of stroke in patients with stroke and transient ischemic attack a guideline for healthcare professionals from the American Heart Association/American Stroke Association. Stroke (2014) 45(7):2160–236. 10.1161/STR.0000000000000024 24788967

[47] O’Sullivan GJ Semba CP Bittner CA Kee ST Razavi MK Sze DY Endovascular management of iliac vein compression (May-Thurner) syndrome. J Vasc Interv Radiol (2000) 11(7):823–36. 10.1016/S1051-0443(07)61796-5 10928517

[48] Furlan AJ Reisman M Massaro J Mauri L Adams H Albers GW Closure or medical therapy for cryptogenic stroke with patent foramen ovale. N Engl J Med (2012) 366(11):991–9. 10.1056/NEJMoa1009639 22417252

[49] Meier B Kalesan B Mattle HP Khattab AA Hildick-Smith D Dudek D Percutaneous closure of patent foramen ovale in cryptogenic embolism. N Engl J Med (2013) 368(12):1083–91. 10.1056/NEJMoa1211716 23514285

[50] Carroll JD Saver JL Thaler DE Smalling RW Berry S MacDonald LA Closure of patent foramen ovale versus medical therapy after cryptogenic stroke. N Engl J Med (2013) 368(12):1092–100. 10.1056/NEJMoa1301440 23514286

[51] Wolfrum M Froehlich GM Knapp G Casaubon LK DiNicolantonio JJ Lansky AJ Stroke prevention by percutaneous closure of patent foramen ovale: a systematic review and meta-analysis. Heart (2013) 100(5):389–95. 10.1136/heartjnl-2013-304394 23793373

[52] Homma S Sacco RL Di Tullio MR Sciacca RR Mohr JP PFO in Cryptogenic Stroke Study (PICSS) Investigators. Effect of medical treatment in stroke patients with patent foramen ovale patent foramen ovale in Cryptogenic Stroke Study. Circulation (2002) 105(22):2625–31. 10.1161/01.CIR.0000017498.88393.44 12045168

[53] Kent DM Dahabreh IJ Ruthazer R Furlan AJ Weimar C Serena J Anticoagulant vs. antiplatelet therapy in patients with cryptogenic stroke and patent foramen ovale: an individual participant data meta-analysis. Eur Heart J (2015) 36(35):2381–9. 10.1093/eurheartj/ehv252 26141397 PMC4568404

[54] Patti G Pelliccia F Gaudio C Greco C. Meta-analysis of net long-term benefit of different therapeutic strategies in patients with cryptogenic stroke and patent foramen ovale. Am J Cardiol (2015) 115(6):837–43. 10.1016/j.amjcard.2014.12.051 25620037

[55] Cheitlin MD Armstrong WF Aurigemma GP Beller GA Bierman FZ Davis JL ACC/AHA/ASE 2003 Guideline for the Clinical Application of Echocardiography (2006). Available from: http://www.ncbi.nlm.nih.gov/pubmed/12952829

[56] Barkhausen J Hunold P Eggebrecht H Schüler WO Sabin GV Erbel R Detection and characterization of intracardiac thrombi on MR imaging. AJR Am J Roentgenol (2002) 179:1539. 10.2214/ajr.179.6.1791539 12438051

[57] Grover S Srinivasan G Selvanayagam JB. Myocardial viability imaging: does it still have a role in patient selection prior to coronary revascularisation? Heart, Lung Circ (2012) 21(8):468–79. 10.1016/j.hlc.2012.03.008 22521496

[58] Kramer CM Barkhausen J Flamm SD Kim RJ Nagel E Standardized cardiovascular magnetic resonance imaging (CMR) protocols, society for cardiovascular magnetic resonance: board of trustees task force on standardized protocols. J Cardiovasc Magn Reson (2008) 10:35 10.1186/1532-429X-10-35 18605997 PMC2467420

[59] Menghetti L Basso C Nava A Angelini A Thiene G. Spin-echo nuclear magnetic resonance for tissue characterisation in arrhythmogenic right ventricular cardiomyopathy. Heart (1996) 76:467. 10.1136/hrt.76.6.467 9014792 PMC484595

[60] Beerbaum P Körperich H Barth P Esdorn H Gieseke J Meyer H Noninvasive quantification of left-to-right shunt in pediatric patients: phase-contrast cine magnetic resonance imaging compared with invasive oximetry. Circulation (2001) 103(20):2476. 11369688 10.1161/01.cir.103.20.2476

[61] Sayad DE Willett DL Bridges WH Chwialkowski M McColl R Payne J Noninvasive quantitation of left ventricular wall thickening using cine magnetic resonance imaging with myocardial tagging. Am J Cardiol (1995) 76:985 10.1016/S0002-9149(99)80280-X 7484849

[62] Olin JW Kaufman JA Bluemke DA Bonow RO Gerhard MD Jaff MR Atherosclerotic Vascular Disease Conference: writing Group IV: imaging Circulation (2004) 109:2626 10.1161/01.CIR.0000128521.02390.72 15173045

[63] Summers RM Andrasko-Bourgeois J Feuerstein IM Hill SC Jones EC Busse MK Evaluation of the aortic root by MRI: insights from patients with homozygous familial hypercholesterolemia. Circulation (1998) 98:509. 10.1161/01.CIR.98.6.509 9714107

[64] Schiebler M Axel L Reichek N Aurigemma G Yeager B Douglas P Correlation of cine MR imaging with two-dimensional pulsed Doppler echocardiography in valvular insufficiency. J Comput Assist Tomogr (1987) 11:627. 10.1097/00004728-198707000-00015 3597886

[65] Pflugfelder PW Landzberg JS Cassidy MM Cheitlin MD Schiller NB Auffermann W Comparison of cine MR imaging with Doppler echocardiography for the evaluation of aortic regurgitation. AJR Am J Roentgenol (1989) 152:729. 10.2214/ajr.152.4.729 2784253

[66] Baher A Mowla A Kodali S Polsani VR Nabi F Nagueh SF Cardiac MRI improves identification of etiology of acute ischemic stroke. Cerebrovasc Dis (2014) 37(4):277–84. 10.1159/000360073 24819735

[67] Harloff A Handke M Reinhard M Geibel A Hetzel A. Therapeutic strategies after examination by transesophageal echocardiography in 503 patients with ischemic stroke. Stroke (2006) 7:859–64. 10.1161/01.STR.0000202592.87021.b7 16439702

[68] Gupta A Gialdini G Lerario MP Baradaran H Giambrone A Navi BB Magnetic resonance angiography detection of abnormal carotid artery plaque in patients with cryptogenic stroke. J Am Heart Assoc (2015) 4:e002012. 10.1161/JAHA.115.002012 26077590 PMC4599540

[69] Srichai MB Junor C Rodriguez LL Stillman AE Grimm RA Lieber ML Clinical, imaging, and pathological characteristics of left ventricular thrombus: a comparison of contrast-enhanced magnetic resonance imaging, transthoracic echocardiography, and transesophageal echocardiography with surgical or pathological validation. Am Heart J (2006) 152:75. 10.1016/j.ahj.2005.08.021 16824834

[70] Weinsaft JW Kim HW Shah DJ Klem I Crowley AL Brosnan R Detection of left ventricular thrombus by delayed-enhancement cardiovascular magnetic resonance prevalence and markers in patients with systolic dysfunction. J Am Coll Cardiol (2008) 52:148–57. 10.1016/j.jacc.2008.03.041 18598895

[71] Freilinger TM Schindler A Schmidt C Grimm J Cyran C Schwarz F Prevalence of nonstenosing, complicated atherosclerotic plaques in cryptogenic stroke. JACC Cardiovasc Imaging (2012) 5:397–405. 10.1016/j.jcmg.2012.01.012 22498329

[72] Molina C Santamarina E Alvarez-Sabín J. Cryptogenic stroke, aortic arch atheroma and patent foramen ovale. Cerebrovasc Dis (2007) 24(Suppl 1):84–8. 10.1159/000107382 17971642

[73] Amarenco P Cohen A Tzourio C Bertrand B Hommel M Besson G Atherosclerotic disease of the aortic arch and the risk of ischemic stroke. N Engl J Med (1994) 331:1474–9. 10.1056/NEJM199412013312202 7969297

[74] Macleod MR Amarenco P Davis SM Donnan GA. Atheroma of the aortic arch: an important and poorly recognised factor in the aetiology of stroke. Lancet Neurol (2004) 3:408–14. 10.1016/S1474-4422(04)00806-3 15207797

[75] Rundek T Di Tullio MR Sciacca RR Titova IV Mohr JP Homma S Association between large aortic arch atheromas and high-intensity transient signals in elderly stroke patients. Stroke (1999) 33:2683–6. 10.1161/01.STR.30.12.2683 10582997

[76] Castellanos M Serena J Segura T Pérez-Ayuso MJ Silva Y Dávalos A. Atherosclerotic aortic arch plaques in cryptogenic stroke: a microembolic signal monitoring study. Eur Neurol (2001) 45:145–50. 10.1159/000052113 11306857

[77] Viguier A Pavy le Traon A Massabuau P Valton L Larrue V. Asymptomatic cerebral embolic signals in patients with acute cerebral ischaemia and severe aortic arch atherosclerosis. J Neurol (2001) 248:768–71. 10.1007/s004150170092 11596781

[78] Donohue KG Saap L Falanga V. Cholesterol crystal embolization: an atherosclerotic disease with frequent and varied cutaneous manifestations. J Eur Acad Dermatol Venereol (2003) 17:504. 10.1046/j.1468-3083.2003.00710.x 12941082

[79] Amarenco P Duyckaerts C Tzourio C Hénin D Bousser MG Hauw JJ. The prevalence of ulcerated plaques in the aortic arch in patients with stroke. N Engl J Med (1992) 326:221–5. 10.1056/NEJM199201233260402 1727976

[80] Jones EF Kalman JM Calafiore P Tonkin AM Donnan GA. Proximal aortic atheroma. An independent risk factor for cerebral ischemia. Stroke (1995) 26:218–24. 7831691

[81] Amarenco P Cohen A Baudrimont M Bousser MG Transesophageal echocardiography detection of aortic arch disease in patients with cerebral infarction. Stroke (1992) 23:1005–9. 10.1161/01.STR.23.7.1005 1615532

[82] Stone DA Hawke MW LaMonte M Kittner SJ Acosta J Corretti M Ulcerated atherosclerotic plaques in the thoracic aorta are associated with cryptogenic stroke: a multiplane transesophageal echocardiographic study. Am Heart J (1995) 130:105–8. 10.1016/0002-8703(95)90243-0 7611098

[83] Di Tullio MR Sacco RL Gersony D Nayak H Weslow RG Kargman DE Aortic atheromas and acute ischemic stroke: a transesophageal echocardiographic study in an ethnically mixed population. Neurology (1996) 46:1560–6. 10.1212/WNL.46.6.1560 8649549

[84] Fisher CM Gore I Okabe N White PD Atherosclerosis of the carotid and vertebral arteries – Extracranial and intracranial. J Neuropathol Exp Neurol (1965) 24:455–76. 10.1097/00005072-196507000-00007

[85] Ito A Sugioka K Matsumura Y Fujita S Iwata S Hanatani A Rapid and accurate assessment of aortic arch atherosclerosis using simultaneous multi-plane imaging by transesophageal echocardiography. Ultrasound Med Biol (2013) 39(8):1337–42. 10.1016/j.ultrasmedbio.2013.03.011 23711502

[86] Tunick PA Krinsky GA Lee VS Kronzon I Diagnostic imaging of thoracic aortic atherosclerosis. AJR Am J Roentgenol (2000) 174:1119 10.2214/ajr.174.4.1741119 10749263

[87] Tenenbaum A Garniek A Shemesh J Fisman EZ Stroh CI Itzchak Y Dual-helical CT for detecting aortic atheromas as a source of stroke: comparison with transesophageal echocardiography. Radiology (1998) 208:153. 10.1148/radiology.208.1.9646807 9646807

[88] Khatri IA Mian N Alkawi A Janjua N Kirmani JF Saric M Catheter-based aortography fails to identify aortic atherosclerotic lesions detected on transesophageal echocardiography. J Neuroimaging (2005) 15:261. 10.1111/j.1552-6569.2005.tb00319.x 15951409

[89] Di Tullio MR Russo C Jin Z Patent Foramen Ovale in Cryptogenic Stroke Study Investigators. Aortic arch plaques and risk of recurrent stroke and death. Circulation (2009) 119:2376–82. 10.1161/CIRCULATIONAHA.108.811935 19380621 PMC2854144

[90] Tunick PA Nayar AC Goodkin GM Mirchandani S Francescone S Rosenzweig BP Effect of treatment on the incidence of stroke and other emboli in 519 patients with severe thoracic aortic plaque. Am J Cardiol (2002) 90:1320–5. 10.1016/S0002-9149(02)02870-9 12480041

[91] Dressler FA Craig WR Castello R Labovitz AJ. Mobile aortic atheroma and systemic emboli: efficacy of anticoagulation and influence of plaque morphology on recurrent stroke. J Am Coll Cardiol (1998) 31:134–8. 10.1016/S0735-1097(97)00449-X 9426031

[92] Ferrari E Vidal R Chevallier T Baudouy M. Atherosclerosis of the thoracic aorta and aortic debris as a marker of poor prognosis: benefit of oral anticoagulants. J Am Coll Cardiol (1999) 33:1317–22. 10.1016/S0735-1097(99)00003-0 10193733

[93] Amarenco P Davis S Jones EF Cohen AA Heiss WD Kaste M Clopidogrel Plus Aspirin Versus Warfarin in Patients With Stroke and Aortic Arch Plaques. Stroke (2014) 45(5):1248–57. 10.1161/STROKEAHA.113.004251 24699050

[94] Smith SC Jr Allen J Blair SN Bonow RO Brass LM Fonarow GC AHA/ACC guidelines for secondary prevention for patients with coronary and other atherosclerotic vascular disease: 2006 update: endorsed by the National Heart, Lung, and Blood Institute. Circulation (2006) 113:2363 10.1161/CIRCULATIONAHA.106.174516 16702489

[95] Nedeltchev K der Maur TA Georgiadis D Arnold M Caso V Mattle HP Ischaemic stroke in young adults: predictors of outcome and recurrence. J Neurol Neurosurg Psychiatry (2005) 76(2):191–5. 10.1136/jnnp.2004.040543 15654030 PMC1739502

[96] Caplan LR. Dissections of brain-supplying arteries. Nat Clin Pract Neurol (2008) 4(1):34–42. 10.1038/ncpneuro0683 18199995

[97] Gilden D Cohrs RJ Mahalingam R Nagel MA. Varicella zoster virus vasculopathies: diverse clinical manifestations, laboratory features, pathogenesis, and treatment. Lancet Neurol (2009) 8:731–40. 10.1016/S1474-4422(09)70134-6 19608099 PMC2814602

[98] Askalan R Laughlin S Mayank S Chan A MacGregor D Andrew M Chickenpox and stroke in childhood: a study of frequency and causation. Stroke (2001) 32:1257–62. 10.1161/01.STR.32.6.1257 11387484

[99] Braun KP Bulder MM Chabrier S Kirkham FJ Uiterwaal CS Tardieu M The course and outcome of unilateral intracranial arteriopathy in 79 children with ischaemic stroke. Brain (2009) 32:544–57. 10.1093/brain/awn313 19039009 PMC2640213

[100] Kang JH Ho JD Chen YH Lin HC. Increased risk of stroke after a herpes zoster attack: a population-based follow-up study. Stroke (2009) 40:3443–8. 10.1161/STROKEAHA.109.562017 19815828

[101] Lin HC Chien CW Ho JD. Herpes zoster ophthalmicus and the risk of stroke: a population-based follow-up study. Neurology (2010) 74:792–7. 10.1212/WNL.0b013e3181d31e5c 20200348

[102] Nagel MA Cohrs RJ Mahalingam R Wellish MC Forghani B Schiller A The *Varicella zoster* virus vasculopathies: clinical, CSF, imaging, and virologic features. Neurology (2008) 70:853–60. 10.1212/01.wnl.0000304747.38502.e8 18332343 PMC2938740

[103] Bushnell CD Goldstein LB. Diagnostic testing for coagulopathies in patients with ischemic stroke. Stroke (2000) 31(12):3067–78. 10.1161/01.STR.31.12.3067 11108774

[104] Chan MY Andreotti F Becker RC Hypercoagulable states in cardiovascular disease. Circulation (2008) 118(22):2286–97. 10.1161/CIRCULATIONAHA.108.778837 19029477

[105] Lane DA Grant PJ Role of hemostatic gene polymorphisms in venous and arterial thrombotic disease. Blood (2000) 95(5):1517–32. 10688804

[106] Hart RG Kanter MC. Hematologic disorders and ischemic stroke. A selective review. Stroke (1990) 21(8):1111–21. 10.1161/01.STR.21.8.1111 2202092

[107] Bushnell C Siddiqi Z Morgenlander JC Goldstein LB. Use of specialized coagulation testing in the evaluation of patients with acute ischemic stroke. Neurology (2001) 56(5):624–7. 10.1212/WNL.56.5.624 11245714

[108] Ruiz-Irastorza G Crowther M Branch W Khamashta MA Antiphospholipid syndrome. The Lancet (2010) 376(9751):1498–509. 10.1016/S0140-6736(10)60709-X 20822807

[109] Marlar RA Gausman JN. Laboratory testing issues for protein C, protein S, and antithrombin. Int J Lab Hematol (2014) 36(3):289–95. 10.1111/ijlh.12219 24750675

[110] Piazza G Thrombophilia testing, recurrent thrombosis, and women’s health. Circulation (2014) 130(3):283–7. 10.1161/CIRCULATIONAHA.113.007664 25024124

[111] Furie KL Kasner SE Adams RJ Albers GW Bush RL Fagan SC Guidelines for the prevention of stroke in patients with stroke or transient ischemic attack a guideline for healthcare professionals from the American Heart Association/American Stroke Association. Stroke (2011) 42(1):227–76. 10.1161/STR.0b013e3181f7d043 20966421

[112] Graus F Rogers LR Posner JB. Cerebrovascular complications in patients with cancer. Medicine (1985) 64(1):16–35. 10.1097/00005792-198501000-00002 3965856

[113] Varki A Trousseau’s syndrome: multiple definitions and multiple mechanisms. Blood (2007) 110(6):1723–9. 10.1182/blood-2006-10-053736 17496204 PMC1976377

[114] Chaturvedi P Singh AP Batra SK. Structure, evolution, and biology of the MUC4 mucin. FASEB J (2008) 22(4):966–81. 10.1096/fj.07-9673rev 18024835 PMC2835492

[115] Butenas S Orfeo T Mann KG. Tissue factor in coagulation Which? Where? When? Arterioscler Thromb Vasc Biol (2009) 29(12):1989–96. 10.1161/ATVBAHA.108.177402 19592470 PMC2783287

[116] Jander S Sitzer M Wendt A Schroeter M Buchkremer M Siebler M Expression of tissue factor in high-grade carotid artery stenosis association with plaque destabilization. Stroke (2001) 32(4):850–4. 10.1161/01.STR.32.4.850 11283381

[117] Bick RL Cancer-associated thrombosis. N Engl J Med (2003) 349(2):109–10. 10.1056/NEJMp030086 12853582

[118] Robert F. The potential benefits of low-molecular-weight heparins in cancer patients. J Hematol Oncol (2010) 3(3):1–12. 10.1186/1756-8722-3-3 20074349 PMC2830957

[119] Caine GJ Stonelake PS Lip GY Kehoe ST. The hypercoagulable state of malignancy: pathogenesis and current debate. Neoplasia (2002) 4(6):465. 10.1038/sj.neo.7900263 12407439 PMC1550339

[120] Kiechl S Matosevic B Willeit J Primary central nervous system lymphoma resulting in stroke and leukoencephalopathy. In: Hayat MA editor. Tumors of the Central Nervous System (Vol. 9), Netherlands: Springer (2012). p. 29–39.

[121] Kurabayashi H Hishinuma A Uchida R Makita S Majima M. Delayed manifestation and slow progression of cerebral infarction caused by polycythemia rubra vera. Am J Med Sci (2007) 333(5):317–20. 10.1097/MAJ.0b013e31805370a9 17505178

[122] Giray S Sarica FB Arlier Z Bal N Recurrent ischemic stroke as an initial manifestation of an concealed pancreatic adenocarcinoma: Trousseau’s syndrome. Chin Med J (2011) 124(4):637–40. 21362297

[123] Seok JM Kim SG Kim JW Chung CS Kim GM Lee KH Coagulopathy and embolic signal in cancer patients with ischemic stroke. Ann Neurol (2010) 68(2):213–9. 10.1002/ana.22050 20695014

[124] Jang H Lee JJ Lee MJ Ryoo S Yoon CH Kim GM Comparison of enoxaparin and warfarin for secondary prevention of cancer-associated stroke. J Oncol (2015) 2015:502089. 10.1155/2015/502089 26064116 PMC4439482

[125] Hong CT Tsai LK Jeng JS. Patterns of acute cerebral infarcts in patients with active malignancy using diffusion-weighted imaging. Cerebrovasc Dis (2009) 28(4):411–6. 10.1159/000235629 19696480

[126] Spector JT Kahn SR Jones MR Jayakumar M Dalal D Nazarian S. Migraine headache and ischemic stroke risk: an updated meta-analysis. Am J Med (2010) 123:612–6. 10.1016/j.amjmed.2009.12.021 20493462 PMC2900472

[127] Schurks M Rist PM Bigal ME Buring JE Lipton RB Kurth T. Migraine and cardiovascular disease: systematic review and meta-analysis. BMJ (2009) 339:b3914. 10.1136/bmj.b3914 19861375 PMC2768778

[128] Bousser MG Conrad J Kittner S de Lignières B MacGregor EA Massiou H Recommendations on the risk if ischaemic stroke associated with use of combined oral contraceptives and hormone replacement therapy in women with migraine. The International Headache Society Task Force on Combined Oral Contraceptives and Hormone Replacement Therapy. Cephalalgia (2000) 20:155–6. 10997767 10.1046/j.1468-2982.2000.00035.x

[129] Etminan M Takkouche B Isorna FC Samii A Risk of ischaemic stroke in people with migraine: systematic review and meta-analysis of observational studies. BMJ (2005) 330:63 10.1136/bmj.38302.504063.8F 15596418 PMC543862

[130] Kurth T Schurks M Logroscino G Buring JE. Migraine frequency and risk of cardiovascular disease in women. Neurology (2009) 73:581–8. 10.1212/WNL.0b013e3181ab2c20 19553594 PMC2731618

[131] Schurks M Buring JE Kurth T Migraine, migraine features, and cardiovascular disease. Headache (2010) 50:1031–40. 10.1111/j.1526-4610.2009.01609.x 20100297 PMC2891199

[132] Li L Schulz UG Kuker W Rothwell PM Oxford Vascular Study. Age-specific association of migraine with cryptogenic TIA and stroke: population-based study. Neurology (2015) 85:1444–51. 10.1212/WNL.0000000000002059 26423431 PMC4631068

[133] Cavestro C Richetta L L’Episcopo MR Pedemonte E Duca S Di Pietrantonj C. Anatomical variants of the circle of Willis and brain lesions in migraineurs. Can J Neurol Sci (2011) 38:494–9. 10.1017/S0317167100011926 21515511

[134] Pietrobon D Moskowitz MA. Pathophysiology of migraine. Annu Rev Physiol (2013) 75:365–91. 10.1146/annurev-physiol-030212-183717 23190076

[135] Lechat P Mas J Lascault G Loron P Theard M Klimczac M Prevalence of patent foramen ovale in patients with stroke. N Engl J Med (1988) 318:1148–52. 10.1056/NEJM198805053181802 3362165

[136] Webster M Chancellor A Smith H Swift D Sharpe D Bass N Patent foramen ovale in young stroke patients. Lancet (1988) 2:11–2. 10.1016/S0140-6736(88)92944-3 2898621

[137] Olesen J Friberg L Olsen T Andersen A Lassen N Hansen P Ischaemia-induced (symptomatic) migraine attacks may be more frequent than migraine-induced ischaemic insults. Brain (1993) 116(Pt 1):187–202. 10.1093/brain/116.1.187 8453456

[138] Dowson A Mullen M Peatfield R Muir K Khan A Wells C Migraine Intervention With STARFlex Technology (MIST) Trial: a prospective, multicenter, double-blinded, sham-controlled trial to evaluate the effectiveness of patent foramen ovale closure with STARFlex septal repair implant to resolve refractory migraine headache. Circulation (2008) 117:1397–404. 10.1161/CIRCULATIONAHA.107.727271 18316488

[139] Tobis J. Management of patients with refractory migraine and PFO: is MIST I relevant? Catheter Cardiovasc Interv (2008) 72:60–4. 10.1002/ccd.21504 18383146

[140] Rundek T Elkind MS Di Tullio MR Carrera E Jin Z Sacco RL Patent foramen ovale and migraine: a cross-sectional study from the Northern Manhattan Study (NOMAS). Circulation (2008) 118:1419–24. 10.1161/CIRCULATIONAHA.108.771303 18794393 PMC2737546

[141] Kruit MC van Buchem MA Launer LJ Terwindt GM Ferrari MD. Migraine is associated with an increased risk of deep white matter lesions, subclinical posterior circulation infarcts and brain iron accumulation: the population-based MRI CAMERA study. Cephalalgia (2010) 30(2):129–36. 10.1111/j.1468-2982.2009.01904.x 19515125 PMC3241741

[142] Headache Classification Committee of the International Headache Society (IHS). The international classification of headache disorders (beta version). Cephalalgia (2013) 33(9):629–808. 10.1177/0333102413485658 23771276

[143] Bousser MG Welch KMA Relation between migraine and stroke. Lancet Neurol (2005) 4(9):533–42. 10.1016/S1474-4422(05)70164-2 16109360

[144] Garg P Servoss SJ Wu JC Bajwa ZH Selim MH Dineen A Lack of association between migraine headache and patent foramen ovale: results of a case-control study. Circulation (2010) 121:1406–12. 10.1161/CIRCULATIONAHA.109.895110 20231534

[145] Davis D Gregson J Willeit P Stephan B Al-Shahi Salman R Brayne C. Patent foramen ovale, ischemic stroke and migraine: systematic review and stratified meta-analysis of association studies. Neuroepidemiology (2013) 40:56–67. 10.1159/000341924 23075508 PMC3707011

[146] Meschia J Bushnell C Boden-Albala B Braun LT Bravata DM Chaturvedi S Guidelines for the primary prevention of stroke: a statement for healthcare professionals from the American Heart Association/American Stroke Association. Stroke (2014) 45:3754–832. 10.1161/STR.0000000000000046 25355838 PMC5020564

[147] Schürks M Rist PM Bigal ME Buring JE Lipton RB Kurth T. Migraine and cardiovascular disease: systematic review and meta-analysis. BMJ (2009) 339:b3914. 10.1136/bmj.b3914 19861375 PMC2768778

[148] Kurth T Schürks M Logroscino G Buring JE. Migraine frequency and risk of cardiovascular disease in women. Neurology (2009) 73(8):581–8. 10.1212/WNL.0b013e3181ab2c20 19553594 PMC2731618

[149] Tietjen GE Khubchandani J. Platelet dysfunction and stroke in the female migraineur. Curr Pain Headache Rep (2009) 13(5):386–91. 10.1007/s11916-009-0063-4 19728966

[150] Tietjen GE Migraine and ischemic heart disease and stroke: potential mechanisms and treatment implications. Cephalalgia (2007) 27:981–7. 10.1111/j.1468-2982.2007.01407.x 17661875

[151] Ridker PM Cook NR Lee IM Gordon D Gaziano JM Manson JE A randomized trial of low-dose aspirin in the primary prevention of cardiovascular disease in women. N Engl J Med (2005) 352:1293–304. 10.1056/NEJMoa050613 15753114

[152] Shi Q Chen J Pongmoragot J Lanthier S Saposnik G Prevalence of Fabry disease in stroke patients – a systematic review and meta-analysis. J Stroke Cerebrovasc Dis (2014) 23(5):985–92. 10.1016/j.jstrokecerebrovasdis.2013.08.010 24126289

[153] Desnick RJ Sweeley CC Fabry’s disease: alpha-galactosidase A deficiency. In: Stanbury JB Wyngaarden JB Fredrickson DS Goldstein JL, editors. Peripheral Neuropathy. New York: McGraw Hill (1983). 2 p.

[154] Eng CM Desnick RJ. Molecular basis of Fabry disease: mutations and polymorphisms in the human alpha-galactosidase A gene. Hum Mutat (1994) 3:103. 10.1002/humu.1380030204 7911050

[155] Eng CM Ashley GA Burgert TS Enriquez AL D’Souza M Desnick RJ. Fabry disease: thirty-five mutations in the alpha-galactosidase A gene in patients with classic and variant phenotypes. Mol Med (1997) 3:174. 9100224 PMC2230047

[156] Topaloglu AK Ashley GA Tong B Shabbeer J Astrin KH Eng CM Twenty novel mutations in the alpha-galactosidase A gene causing Fabry disease. Mol Med (1999) 5:806. 10666480 PMC2230489

[157] Wolters FJ Rinkel GJ Vergouwen MD. Clinical course and treatment of vertebrobasilar dolichoectasia: a systematic review of the literature. Neurol Res (2013) 35(2):131–7. 10.1179/1743132812Y.0000000149 23452575

[158] Saarto T Wiffen PJ. Antidepressants for neuropathic pain. Cochrane Database Syst Rev (2005) 3:CD005454. 16034979 10.1002/14651858.CD005454

[159] Moore DF Scott LT Gladwin MT Altarescu G Kaneski C Suzuki K Regional cerebral hyperperfusion and nitric oxide pathway dysregulation in Fabry disease: reversal by enzyme replacement therapy. Circulation (2001) 104:1506. 10.1161/hc3801.096352 11571244

[160] Sher NA Letson RD Desnick RJ The ocular manifestations in Fabry’s disease. Arch Ophthalmol (1979) 97:671 10.1001/archopht.1979.01020010327008 106811

[161] Maisey DN Cosh JA. Basilar artery aneurysm and Anderson-Fabry disease. J Neurol Neurosurg Psychiatry (1980) 43:85. 10.1136/jnnp.43.1.85 6766499 PMC490469

[162] Mitsias P Levine SR Cerebrovascular complications of Fabry’s disease. Ann Neurol (1996) 40:8 10.1002/ana.410400105 8687196

[163] Garzuly F Maródi L Erdös M Grubits J Varga Z Gelpi E Megadolichobasilar anomaly with thrombosis in a family with Fabry’s disease and a novel mutation in the alpha-galactosidase A gene. Brain (2005) 128:2078 10.1093/brain/awh546 15947062

[164] Buechner S Moretti M Burlina AP Cei G Manara R Ricci R Central nervous system involvement in Anderson-Fabry disease: a clinical and MRI retrospective study. J Neurol Neurosurg Psychiatry (2008) 79:1249. 10.1136/jnnp.2008.143693 18535022

[165] MacDermot KD Holmes A Miners AH Anderson-Fabry disease: clinical manifestations and impact of disease in a cohort of 60 obligate carrier females. J Med Genet (2001) 38:769 10.1136/jmg.38.11.750 11732485 PMC1734754

[166] Branton MH Schiffmann R Sabnis SG Murray GJ Quirk JM Altarescu G Natural history of Fabry renal disease: influence of alpha-galactosidase A activity and genetic mutations on clinical course. Medicine (Baltimore) (2002) 81:122 10.1097/00005792-200203000-00003 11889412

[167] Yam GH Zuber C Roth J. A synthetic chaperone corrects the trafficking defect and disease phenotype in a protein misfolding disorder. FASEB J (2005) 19:12. 10.1096/fj.04-2375com 15629890

[168] Maizel SE Simmons RL Kjellstrand C Fryd DS Ten-year experience in renal transplantation for Fabry’s disease. Transplant Proc (1981) 13:57. 6791331

[169] Ojo A Meier-Kriesche HU Friedman G Hanson J Cibrik D Leichtman A Excellent outcome of renal transplantation in patients with Fabry’s disease. Transplantation (2000) 69:2337 10.1097/00007890-200006150-00020 10868636

[170] Shah T Gill J Malhotra N Takemoto SK Bunnapradist S. Kidney transplant outcomes in patients with Fabry disease. Transplantation (2009) 87:280. 10.1097/TP.0b013e318191a842 19155985

[171] Germain DP Charrow J Desnick RJ Guffon N Kempf J Lachmann RH Ten-year outcome of enzyme replacement therapy with agalsidase beta in patients with Fabry disease. J Med Genet (2015) 52(5):353–8. 10.1136/jmedgenet-2014-102797 25795794 PMC4413801

[172] Karttunen V Alfthan G Hiltunen L Rasi V Kervinen K Kesäniemi YA Risk factors for cryptogenic ischaemic stroke. Eur J Neurol (2002) 9:625–32. 10.1046/j.1468-1331.2002.00464.x 12453078

[173] Parnetti L Caso V Santucci A Corea F Lanari A Floridi A Mild hyperhomocysteinemia is a risk-factor in all etiological subtypes of stroke. Neurol Sci (2004) 25:13–7. 10.1007/s10072-004-0219-5 15060810

[174] Ribó M Montaner J Monasterio J Molina C Arenillas J Chaócnand P Papeldelahomocisteínaen la fase hiperaguda del ictus. Neurologia (2004) 19:10. 14762728

[175] Vayá A Ejarque I Tembl J Corella D Laiz B. Hyperhomocysteinemia, obesity and cryptogenic stroke. Clin Hemorheol Microcirc (2011) 47:53–8. 10.3233/CH-2010-1365 21321408

[176] Eikelboom JW Hankey GJ Anand SS Lofthouse E Staples N Baker RI. Association between high homocyst(e)ine and ischemic stroke due to large- and small-artery disease but not other etiologic subtypes of ischemic stroke. Stroke (2000) 31:1069–75. 10.1161/01.STR.31.5.1069 10797167

[177] Hirsch S Poniachick J Avendaño M Csendes A Burdiles P Smok G Serum folate and homocysteine levels in obese females with non-alcoholic fatty liver. Nutrition (2005) 21:137–41. 10.1016/j.nut.2004.03.022 15723740

[178] Karatela RA Sainani GS. Plasma homocysteine in obese, overweight and normal weight hypertensives and normotensives. Indian Heart J (2009) 61:156–9. 20039500

[179] Narin F Atabek ME Karakukcu M Narin N Kurtoglu S Gumus H The association of plasma homocysteine levels with serum leptin and apolipoprotein B levels in childhood obesity. Ann Saudi Med (2005) 25:209–14. 16119521 10.5144/0256-4947.2005.209 PMC6147986

[180] Boushey CJ Beresford SA Omenn GS Motulsky AG A quantitative assessment of plasma homocysteine as a risk factor for vascular disease: probable benefits of increasing folic acid intakes. JAMA (1995) 274:1049–57. 10.1001/jama.1995.03530130055028 7563456

[181] Klatsky AL Armstrong MA Friedman GD Sidney S. Alcohol drinking and risk of hemorrhagic stroke. Neuroepidemiology (2002) 21:115–22. 10.1159/000054808 12006774

[182] Welch GN Loscalzo J Homocysteine and atherothrombosis. N Engl J Med (1998) 338:1042–50. 10.1056/NEJM199804093381507 9535670

[183] Selhub J Jacques PF Wilson PW Rush D Rosenberg IH. Vitamin status and intake as primary determinants of homocysteinemia in an elderly population. JAMA (1993) 270:2693–8. 10.1001/jama.1993.03510220049033 8133587

[184] Lee M Hong KS Chang SC Saver JL. Efficacy of homocysteine-lowering therapy with folic acid in stroke prevention: a meta-analysis. Stroke (2010) 41:1205–12. 10.1161/STROKEAHA.109.573410 20413740 PMC2909661

[185] Lonn E Yusuf S Arnold MJ Sheridan P Pogue J Micks M Heart outcomes prevention evaluation (HOPE) 2 investigators. Homocysteine lowering with folic acid and B vitamins in vascular disease. N Engl J Med (2006) 354:1567–77. 10.1056/NEJMoa060900 16531613

[186] Ballabio E Bersano A Bresolin N Candelise L. Monogenic vessel diseases related to ischemic stroke: a clinical approach. J Cereb Blood Flow Metab (2007) 27(10):1649–62. 10.1038/sj.jcbfm.9600520 17579657

[187] Hassan A Sham PC Markus HS. Planning genetic studies in human stroke Sample size estimates based on family history data. Neurology (2002) 58(10):1483–8. 10.1212/WNL.58.10.1483 12034783

[188] Johnson MW Thomley ML Huang SS Gass JD. Idiopathic recurrent branch retinal arterial occlusion. Natural history and laboratory evaluation. Ophthalmology (1994) 101(3):480–9. 10.1016/S0161-6420(94)31309-1 8127568

[189] Gass JD Tiedeman J Thomas MA. Idiopathic recurrent branch retinal arterial occlusion. Ophthalmology (1986) 93(9):1148–57. 10.1016/S0161-6420(86)33600-5 3808626

[190] Susac JO Hardman JM Selhorst JB Microangiopathy of the brain and retina. Neurology (1979) 29:313–6. 10.1212/WNL.29.3.313 571975

[191] Futrell N Millikan C. Frequency, etiology, and prevention of stroke in patients with systemic lupus erythematosus. Stroke (1989) 20:583. 10.1161/01.STR.20.5.583 2718197

[192] Mikdashi J Handwerger B Langenberg P Miller M Kittner S. Baseline disease activity, hyperlipidemia, and hypertension are predictive factors for ischemic stroke and stroke severity in systemic lupus erythematosus. Stroke (2007) 38:281. 10.1161/01.STR.0000254476.05620.14 17218611

[193] Krishnan E. Stroke subtypes among young patients with systemic lupus erythematosus. Am J Med (2005) 118:1415. 10.1016/j.amjmed.2005.05.026 16378793

[194] Mitsias P Levine SR. Large cerebral vessel occlusive disease in systemic lupus erythematosus. Neurology (1994) 44:385. 10.1212/WNL.44.3_Part_1.385 8145903

[195] Liang MH Karlson E Neurologic manifestations of lupus. 2nd ed In: Schur PH, editor. The Clinical Management of Systemic Lupus Erythematosus. Philadelphia: Lippincott (1996).

[196] Roldan CA Gelgand EA Qualls CR Sibbitt WL Jr. Valvular heart disease as a cause of cerebrovascular disease in patients with systemic lupus erythematosus. Am J Cardiol (2005) 95:1441–7. 10.1016/j.amjcard.2005.02.010 15950567

[197] Nouh A Carbunar O Ruland S Neurology of rheumatologic disorders. Curr Neurol Neurosci Rep (2014) 14(7):1–13. 10.1007/s11910-014-0456-6 24871965

[198] Clinicaltrials. (2016). Available from: https://clinicaltrials.gov/ct2/show/NCT02239120 Year of public Query

[199] Population Health Research Institute. (2014–2018). Available from: http://www.phri.ca/research/stroke-cognition/navigate-esus/

[200] Marder VJ Chute DJ Starkman S Abolian AM Kidwell C Liebeskind D Analysis of thrombi retrieved from cerebral arteries of patients with acute ischemic stroke. Stroke (2005) 37(8):2086–93. 10.1161/01.STR.0000230307.03438.94 16794209

[201] Kim SK Yoon W Kim TS Kim HS Heo TW Park MS. Histologic analysis of retrieved clots in acute ischemic stroke: correlation with stroke etiology and gradient-echo MRI. AJNR Am J Neuroradiol (2015) 36(9):1756–62. 10.3174/ajnr.A4402 26159515 PMC7968760

[202] Santamarina E Penalba A García-Berrocoso T Delgado P Quintana M González-Alujas T Biomarker level improves the diagnosis of embolic source in ischemic stroke of unknown origin. J Neurol (2012) 259(12):2538–45. 10.1007/s00415-012-6532-4 22592287

[203] Muuronen AT Taina M Hedman M Marttila J Kuusisto J Onatsu J Increased visceral adipose tissue as a potential risk factor in patients with Embolic Stroke of Undetermined Source (ESUS). PLoS One (2015) 10(3):e0120598. 10.1371/journal.pone.0120598 25756793 PMC4354901

[204] Blasi F Oliveira BL Rietz TA Rotile NJ Naha PC Cormode DP Multisite thrombus imaging and fibrin content estimation with a single whole-body PET scan in rats. Arterioscler Thromb Vasc Biol (2015) 35(10):2114–21. 10.1161/ATVBAHA.115.306055 26272938 PMC4583361

